# Long-term B cell memory emerges at uniform relative rates in the human immune response

**DOI:** 10.1073/pnas.2406474122

**Published:** 2025-02-28

**Authors:** Ivana Cvijović, Michael Swift, Stephen R. Quake

**Affiliations:** ^a^Department of Applied Physics, Stanford University, Stanford, CA 94305; ^b^Department of Chemical and Systems Biology, Stanford University, Stanford, CA 94305; ^c^Department of Bioengineering, Stanford University, Stanford, CA 94305; ^d^The Chan Zuckerberg Initiative, Redwood City, CA 94063

**Keywords:** BCR, long lived plasma cells, dynamical models, systems biology

## Abstract

B cells generate pathogen-specific antibodies and play an essential role in providing adaptive protection against infection. Antibody genes are modified in evolutionary processes acting on the B cell populations within an individual. These populations proliferate, differentiate, and migrate to long-term niches in the body. However, the dynamics of these processes in the human immune system are primarily inferred from mouse studies. We addressed this gap by sequencing the antibody repertoire and transcriptomes from single B cells in four immune-rich tissues from six individuals. We find that B cells descended from the same pre-B cell (“lineages”) often colocalize within the same tissue, with the bone marrow harboring the largest excess of lineages without representation in other tissues. Within lineages, cells with different levels of somatic hypermutation are uniformly distributed among tissues and functional states. This suggests that the relative probabilities of localization and differentiation outcomes change negligibly during affinity maturation, and quantitatively agrees with a simple dynamical model of B cell differentiation. While lineages strongly colocalize, we find individual B cells nevertheless appear to make independent differentiation decisions. Proliferative antibody-secreting cells, however, deviate from these global patterns. These cells are often clonally expanded, their clones appear universally distributed among all sampled organs, and form lineages with an excess of cells of the same type. Collectively, our findings show the limits of peripheral blood monitoring of the immune repertoire, and provide a probabilistic model of the dynamics of antibody memory formation in humans.

## Significance

Antibodies created during an encounter with a pathogen can be retained in molecular memory for decades, offering lifetime protection. Despite numerous studies that investigate the human antibody response in the blood, we still lack an understanding of the dynamics of long-term antibody memory formation. This is partly because cells that produce antibodies committed to long-term memory live in the bone marrow, which is difficult to sample in humans. By sampling antibody-producing cells in the bone marrow and other immune-rich tissues and analyzing their genetic relationships, we infer a quantitative model of the dynamics of antibody memory formation. Our findings reveal that long-term antibody memory is created routinely and uniformly during immune response, challenging earlier results.

The human antibody response provides protection against pathogens. The stability of this protective memory varies widely between pathogens, vaccines, and individuals, lasting for periods of times ranging from months to decades (1). The durability of antibody memory is determined by the creation and persistence of the B cells that produce antibodies and is of great interest for vaccine development (2–4). However, much about the dynamical process that generates durable antibody memory in humans remains poorly understood.

Human B cells exist in many different functional states or cell types, which have been characterized through immunophenotyping and single-cell sequencing studies (5–9). These states differ in their ability to survive, secrete, and further evolve their antibodies in future immune responses. There are two major B cell memory states generated in immune responses: memory B cells and long-lived antibody-secreting cells (ASCs). Memory B cells persist for long periods of time without secreting antibodies but can rapidly proliferate and differentiate into ASCs when re-exposed to pathogens (10, 11). Such ASCs are often referred to as plasmablasts, are thought to be recently activated, and are believed to be short-lived. In contrast, long-lived ASCs are terminally differentiated and constantly secrete antibodies independent of the time since pathogen encounter (12, 13). Unlike memory B cells, they are rarely found circulating in the peripheral blood and are thought to primarily reside in the bone marrow (14). Both Memory B cells and ASCs are generated from naive B cells in microanatomical structures known as germinal centers that arise during an immune response (15).

Work in model organisms has described the process of affinity maturation and B cell differentiation in the germinal center in great detail (15). Generally, in an immune response, germinal centers are seeded with what can be a large number of unique B cell clones (15). Clones with antibodies that bind the antigen proliferate and diversify their antibody nucleotide sequences via random mutations, creating a lineage of genetically distinguishable related cells. As the immune response proceeds, B cells compete on the basis of the affinity of each cell’s B cell receptor (membrane-bound form of antibody) to receive proliferation and differentiation signals from T cells. Differences in these signals direct members of each lineage to become short or long-lived ASCs or memory B cells (15, 16). A similar set of signals directs these cells to their particular long-term niches, such as the bone marrow, after leaving germinal centers (14).

In humans, less is known about the temporal dynamics of affinity maturation and how they determine the timing of differentiation and proliferation signals within an immune response. For instance, it is not known when in the course of affinity maturation lineages produce long-lived ASCs or memory B cells, and to what extent differentiation and localization signals are similar for members of the same lineage. In mice, there remain two opposing paradigms for the pace of differentiation in the germinal center reaction (15). Some lines of research provide support for a temporal switch in the germinal center reaction, in which memory B cells are favored early in the germinal center response and ASCs arise preferentially later (17, 18). Other work has provided evidence that the generation of long-lived ASCs and the localization of these populations to the bone marrow is uniform in time during an immune response (19, 20). It is not clear to what degree either of these paradigms developed in mouse models applies to humans, where analogous measurements of long-term memory populations in the same healthy individual over time would be highly invasive. Antibody repertoire sequencing can provide a statistical view of human B cell evolution and memory formation, which can allow the identification of dynamical processes consistent with such statistics. For example, repertoire sequencing has identified the genetic relationships between B cells in the blood (21), during vaccination (22–24), the order and pace of class-switch recombination (25), and the genetic relationships between B cells in different tissues (23, 26, 27). Despite these advances, understanding the dynamics of long-term antibody memory formation in healthy humans has been challenging. Some insight into the dynamics of memory maintenance exists from a study that has investigated the persistence of human bone marrow plasma cells through serial sampling of remitting childhood cancer patients (28), or from focused measurements in healthy human influenza vaccine recipients (29). However, repertoire-wide insight into the dynamics of long-term memory formation in healthy individuals has been limited.

In principle, the dynamics of long-term memory formation may be inferred from a single time point using the genetic relationships of B cells of different types. B cells descended from the same recombination event carry a unique genetic recombination signature that allows the identification of related B cells from their antibody sequences. Moreover, since hypermutation of antibody sequences occurs primarily in the germinal center reaction (15), hypermutations represent a molecular marker of events that occurred during affinity maturation that can be measured in differentiated B cells long after they have emerged from the germinal center. Differences in the dynamical process in the germinal center reaction thus lead to differences in the statistical patterns of antibody repertoires. For example, the temporal switch model and the uniform emergence model for the dynamics of differentiation in the germinal center predict different relative amounts of hypermutation between memory B cells and ASCs. These and other dynamical processes can be resolved from a single time point as long as we can relate the dynamical process to repertoire statistics via a dynamical model.

The interpretation of repertoire statistics in light of the dynamical process underlying long-term memory formation would require a measurement of the antibody sequences of sufficiently large numbers of B cells in different long-term memory states. Such datasets do not exist because previous work has often either exclusively measured long-lived ASCs (28), could not associate antibody sequences with cell states, or was not sufficiently powered for this type of analysis (6, 7, 30). This gap leaves it impossible to systematically understand, for example, when long-lived ASCs arise during an immune response and how they are related to the B cells that circulate in the blood.

Here, we use paired single-cell transcriptome and Variable, Diversity, and Joining (VDJ) sequencing to measure the phenotypic and genetic relationships of B cells in four immune-rich tissues in six healthy individuals. We then use these measurements to investigate the repertoire-wide patterns of B cell evolution, differentiation, and migration between organs. We infer that B cells make independent differentiation decisions at constant relative rates throughout the course of affinity maturation. Thus, long-lived ASCs are routinely generated at any point in immune responses. Furthermore, groups of related cells have highly correlated localization outcomes, meaning that tissue-resident cells are often found in large lineages that are not observed in the peripheral blood.

## The Distribution of B Cells in Lymphatic Tissues at Rest.

**Fig. 1.**
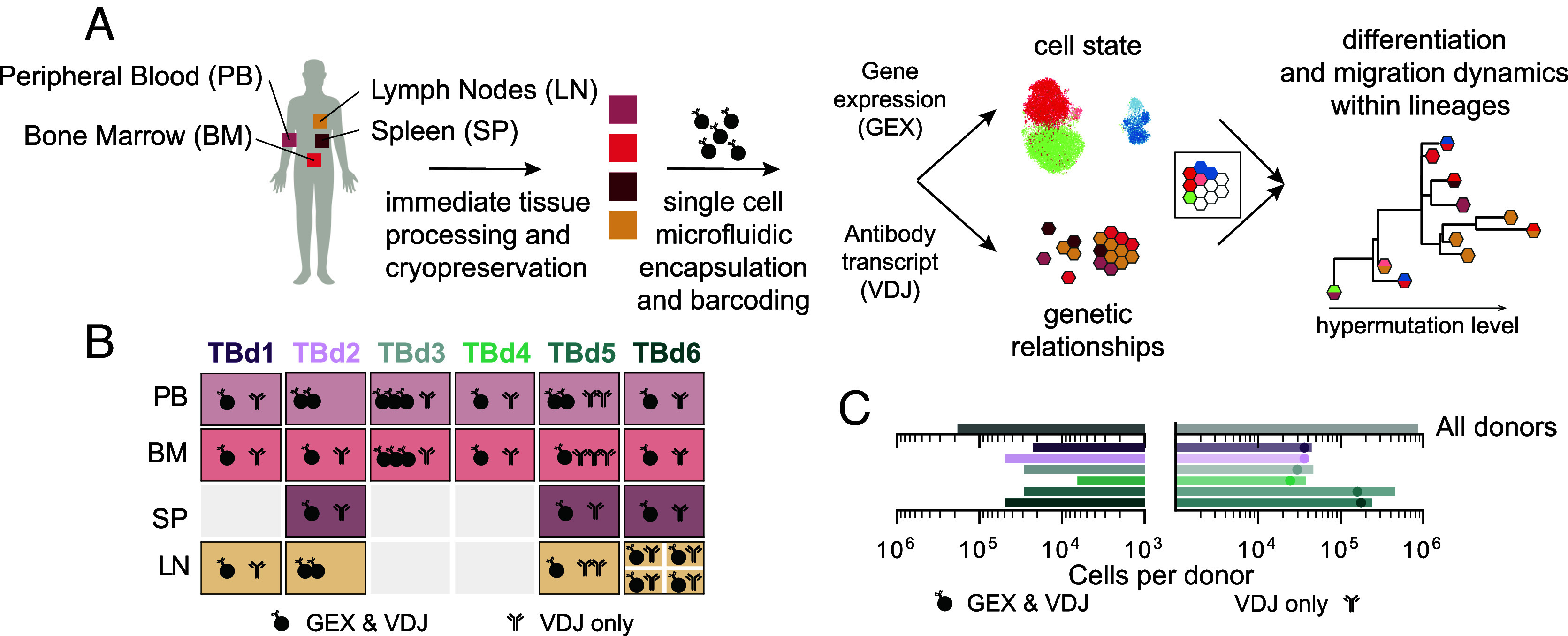
Overview of the study. (*A*) Study design. (*B*) Graphic depicting the data collected for each tissue and donor. GEX denotes single-cell transcriptome sequencing and VDJ denotes antibody transcript (or, equivalently, the B cell receptor (VDJ)) sequencing. Note that for donor TBd6, we collected samples for four separate lymph nodes: three supradiaphragmatic lymph nodes and one mesenteric lymph node. TBd2 and TBd5 lymph node samples were all derived from a single, individually processed supradiaphragmatic lymph node, whereas TBd1 lymph node samples represent the pooled mixture of three mesenteric lymph nodes. (*C*) Total numbers of cells in transcriptionally profiled samples (*Left*) and single-cell VDJs samples (*Right*; see *SI Appendix*, sections B and C for a description of our cell calling algorithms). Dots represent the total number of unique VDJs in single-cell VDJ-only samples.

From six organ donors with no clinical history of cancer, immune disease, or active infectious disease at the time of death, we purified B cells from the vertebral bodies, supradiaphragmatic and mesenteric lymph nodes, the spleen, and the peripheral blood (Fig. 1, Table 1, and *Materials and Methods*). Incidentally, one of our donors, TBd1, had received a Moderna COVID-19 booster within 30 d prior to tissue recovery. We used droplet-based single-cell sequencing to analyze the transcriptomes and antibody sequences (VDJs) of 200 thousand single B cells. To increase the depth of VDJ sampling, and to provide replication of the measurement process, we used the same platform to sequence only the VDJs of an additional 600 thousand B cells.

**Table 1.** Demographic characteristics of donors

Donor	Age	Sex	Ethnicity	Cause of death
TBd1	46	F	Asian/Japanese	Cerebrovascular stroke
TBd2	35	M	American Indian or Alaska Native	Anoxia
TBd3	25	F	Hispanic/Latino	Head Trauma
TBd4	43	F	Hispanic/Latino	Anoxia
TBd5	45	F	Asian/Filipino	Cerebrovascular stroke
TBd6	60	M	Asian/Filipino	Anoxia, spontaneous cardiac arrest

Overall, we observe few B cells with transcriptional phenotypes suggestive of recent germinal center exposure in any donor, consistent with an immune system at rest (*SI Appendix*, Fig. S1). Germinal center B cells were also not more abundant in donor TBd1, the recent recipient of the COVID-19 booster, consistent with ongoing affinity maturation in this donor remaining localized to the draining lymph nodes (31). A small fraction of all B cells (1 to 6% per donor) appear to be proliferating as evidenced by the expression of M-phase markers (*SI Appendix*, Fig. S2 and section B.3). Proliferating cells are predominantly ASCs, though we sampled a small number of proliferating germinal center B cells, as well as a small fraction of atypical B cells (9) that appear to be actively proliferating (*SI Appendix*, Fig. S2*A*). The distribution of B cell subtypes among the sampled organs was consistent with previous findings (7): The secondary lymphatic organs contained an abundance of memory B cells relative to other sampled organs, and the bone marrow was enriched for antibody-secreting cells (*SI Appendix*, Fig. S1).

## Related B Cells Tend to Reside in the Same Tissue.

**Fig. 2.**
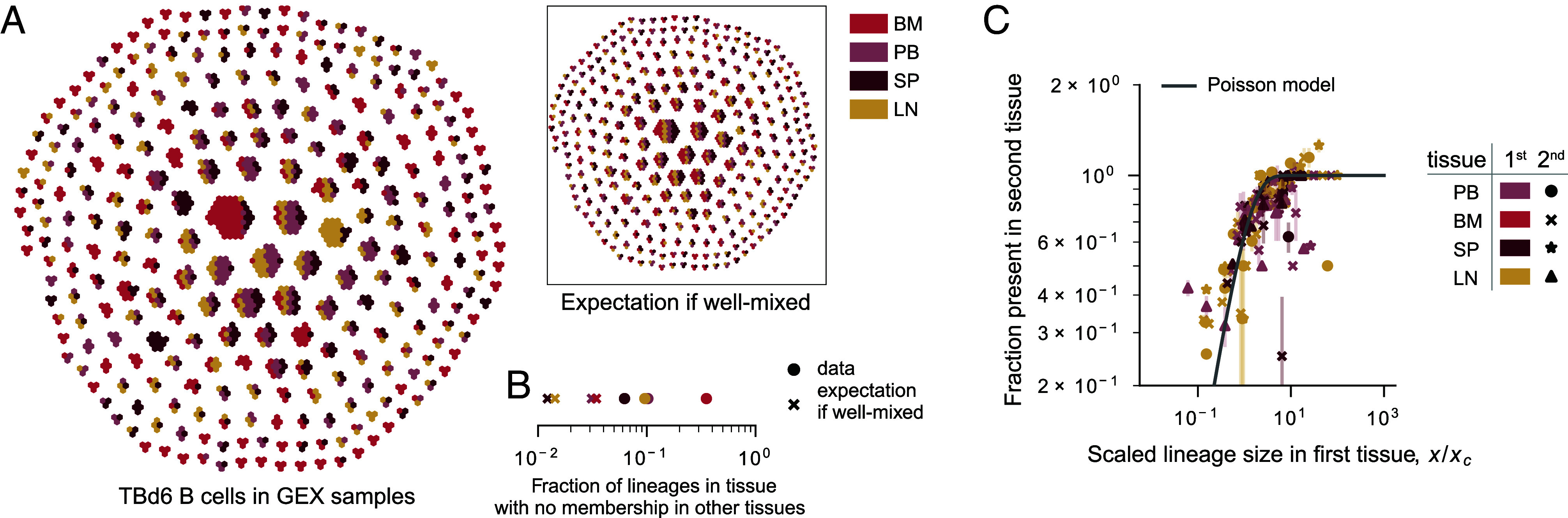
Tissue distribution of B cells descended from the same VDJ recombination event (lineages). (*A*) The tissue composition of lineages from TBd6. Each hexagon denotes a cell, and each connected group of hexagons represents a lineage. Cells are colored by tissue. Each tissue has been downsampled to the same overall number of cells. The *Inset* shows the expectation if the lineages were well mixed among the organs, obtained by randomly permuting the tissue labels among the cells. Lineages are ordered spirally by size, with the largest lineages in the middle. (*B*) The fraction of lineages in a tissue without sampled members in other tissues. Crosses denote well-mixed expectation, obtained by permutation. (*C*) The probability that a lineage is discovered in a tissue, as a function of the total size of that lineage in another, ascertainment, tissue. Points are colored by ascertainment tissue, and symbols denote the second tissue. Size, *x*, represents the total fraction of unique heavy chain sequences attributable to the lineage, and is scaled by a single parameter per pair of tissues, the critical size *x*_*c*_ (see also *SI Appendix*, Fig. S27 for the same data split by tissue and *SI Appendix*, Fig. S3 for the same data split by donor). As described in *SI Appendix*, section E.1, we inferred *x*_*c*_ by fitting the expected curve for a Poisson process, $1-e^{-x/x_{c}}$, to the data. The inferred parameters for each pair of tissues are shown in *SI Appendix*, Fig. S3*D*. As for Fig. 6, all independently generated 10X emulsions from a cell suspension have been downsampled to an equivalent number of unique VDJs per emulsion (5,000), smaller samples were dropped from the analysis, and error bars represent SEs among the same quantity calculated using all available independent pairs of emulsions. The gray line denotes expectation if changes in localization are a Poisson process, $1-e^{-x/x_{c}}$.

B cells descended from the same VDJ recombination event carry a unique genetic recombination signature that we used to group B cells into lineages (*SI Appendix*, section C.1). We found that cells belonging to the same lineage tend to colocalize within the same tissue (Fig. 2 *A* and *B*). This suggests that related B cells receive consistent localization signals. In all donors except TBd1 (*SI Appendix*, Fig. S3*B*), this colocalization is particularly strong in the case of the bone marrow (Fig. 2*B*).

However, B cell colocalization is not absolute: In large lineages, a minority of cells can be found in a different organ. This sharing follows a simple pattern: The probability that a lineage sampled in the blood, the spleen, or a lymph node, is present in a second tissue increases with its size (Fig. 2*C* and *SI Appendix*, Fig. S27). The functional form of this increase suggests that the migration of B cells out of these tissues is governed by a Poisson process, where there is a small, but constant probability that a cell in a lineage found in one of these three tissues localizes in a different tissue. Once again, we note a departure from this overall pattern in TBd1. In this donor, we observe unusually large lineages and unusually high levels of lineage sharing between the bone marrow and the blood (*SI Appendix*, Fig. S3 *A* and *B*), perhaps related to a transient process associated with their recent COVID-19 booster.

## Hypermutation Level Within a Lineage Does Not Predict Tissue Localization.

**Fig. 3.**
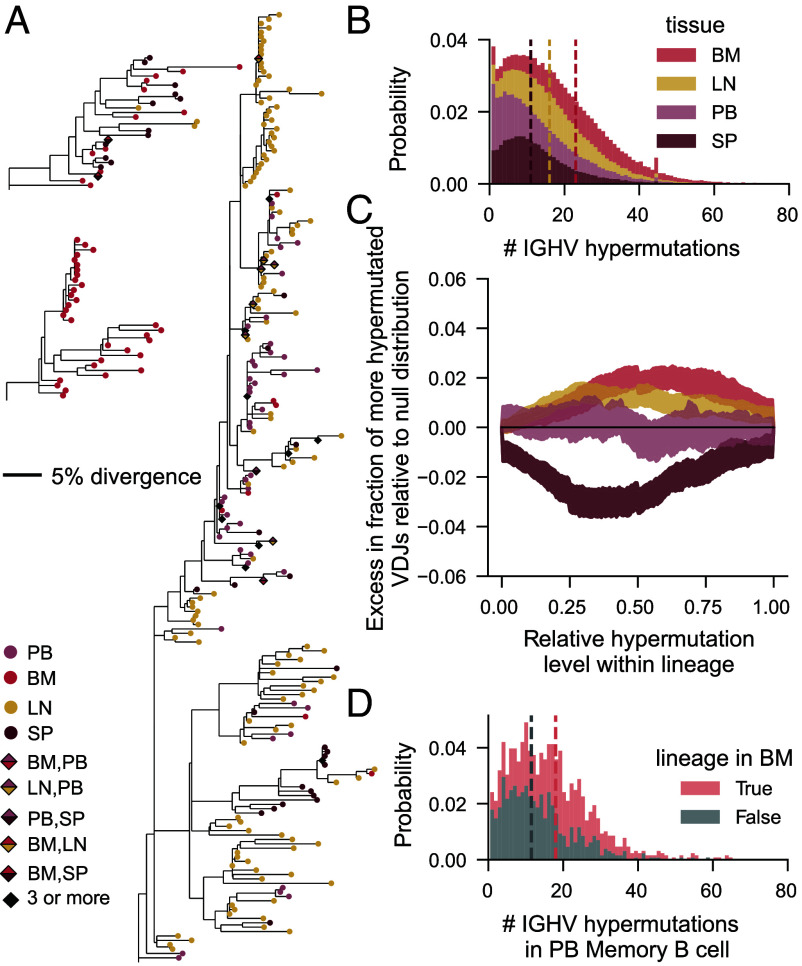
Distribution of hypermutation levels among related cells found in different tissues. (*A*) Templated V gene trees for 3 example lineages from TBd6. Trees are rooted on the inferred germline V sequence. Leaves correspond to unique templated V sequences and are colored by localization. The bar denotes a divergence of 5%. (*B*) Distribution of the number of hypermutations observed in the IGHV gene for all unique heavy chains, split by tissue. Prior to quantifying the distribution, all tissues were downsampled to the same number of unique VDJ sequences. Within each bar, the relative height of a color represents the proportion of the bar attributable to the VDJ sequences from that tissue. Dotted lines represent medians, which overlap for blood and spleen in our data. (*C*) The within-lineage distribution of hypermutated cells in a particular tissue. Lineages with at least 5 unique VDJs are included in this analysis (corresponding to a total of 9,172 lineages across the dataset). For each unique VDJ, we quantified its relative hypermutation level within the lineage: 0 and 1 correspond to the least and most hypermutated sequences in the lineage, respectively. We obtained null distributions of this quantity by permuting tissue labels between hypermutated VDJs within lineages. The CIs represent the bounds on the cumulative density function obtained in 100 independent permutations. We plot the difference between the measured and null distributions. Black denotes perfect agreement. (*D*) Distribution of the number of hypermutations observed in the templated portion of the IGHV gene for VDJs found to be associated in peripheral blood memory B cells. Gray and salmon represent memory B cells with and without a sampled relative in the bone marrow, respectively. Prior to quantifying the distribution, both groups of cells were downsampled to the same number of unique VDJ sequences. Within each bar, the relative height of a color represents the proportion of the bar attributable to each group of cells.

Overall, the above results suggest that B cells belonging to the same lineage get consistent localization signals at the point of germinal center exit, but that a portion of the lineage can still localize in a different tissue. We wanted to understand whether there is covariation between hypermutation and tissue localization (Fig. 3). Such covariation could, for example, be mediated by antibody affinity for antigen, which both increases with hypermutation, and affects cell differentiation potential: Cells with the highest affinity are thought to become ASCs (14, 15), a cell type that is enriched in the bone marrow (*SI Appendix*, Fig. S1).

Notably, the overall level of hypermutation does vary between cells residing in different tissues, and B cells located in bone marrow were, by far, the most hypermutated (Fig. 3*B*). Surprisingly, within lineages, cells sampled in different tissues were almost entirely uniformly distributed in hypermutation level (Fig. 3*C*). Specifically, bone marrow localized cells could appear at any point in the lineage and were only weakly enriched among the most strongly hypermutated cells—the percentage of bone marrow cells that have a relative hypermutation level greater than 0.5 deviates from the null distribution by only on the order of 1%. This apparent incongruity is resolved by the observation that hypermutation levels covary between B cells in the same lineage.

**Fig. 4.**
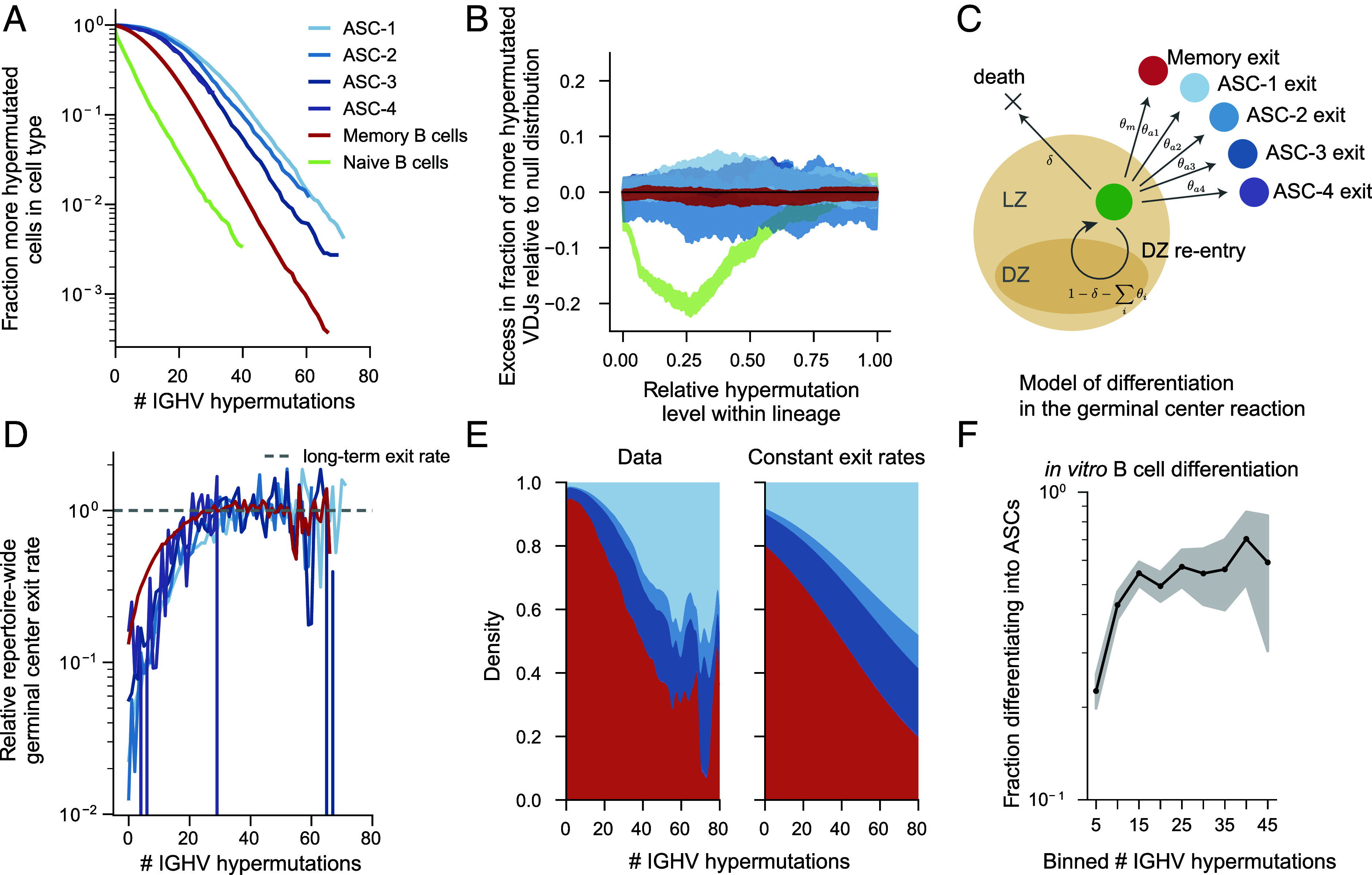
Distribution of hypermutation levels among related cells of different cell types. (*A*) Distribution of the number of IGHV hypermutations for heavy chains associated with different cell types. Cells with no observed hypermutations are excluded, for consistency with the following panels. (*B*) The within-lineage distribution of hypermutated cells in a particular cell type, equivalently to Fig. 3*C*. (*C*) Schematic of a simple model of differentiation in the germinal center reaction in which germinal center B cell make cell fate decisions at rates that do not change as the germinal center reaction proceeds. For each cell fate outcome, the per-cycle rates are denoted in symbols adjacent to the arrows. (*D*) Repertoire-wide effective germinal center exit rates scaled by the relevant long-term germinal center rates for each cell type (*SI Appendix*, section F.2 and Table S3). (*E*) Fraction of cells of each cell type as a function of the hypermutation level. The *Left* panel represents the empirical distribution, and the *Right* represents the distribution expected assuming constant per-cycle exit rates equal to the long-term exit rates for each cell type. The total abundances of B cells of each type were set to be equal to the total abundances in the data. Colors as in previous panels. (*F*) Differentiation statistics of human B cells in vitro: probability of differentiating into an ASC across hypermutation levels reanalyzing in vitro data from ref. 32. Shaded areas denote the 95% CIs and are binomial proportion intervals calculated using the Clopper-Pearson method and bins containing fewer than 10 cells are omitted.

Thus, cells with highly hypermutated relatives are more likely to themselves be more hypermutated. As a corollary of that, if a lineage is detected in the bone marrow (and thus contains highly hypermutated cells), members of that lineage not present in the bone marrow are more hypermutated than comparable cells in the same nonmarrow tissue(Fig. 4*D*). Thus, tissue-level differences in hypermutation in the human antibody repertoire appear to arise from the nonuniform localization of lineages to tissues rather than the nonuniform assortment of cells within a lineage.

The results in Fig. 3*C* suggest that probabilities of localization in a certain tissue change only weakly as hypermutations accumulate over the course of affinity maturation in a lineage. However, tissues are mixtures of B cells of different cell types. Thus, conditioning on tissue presence of a lineage still recovers a mixture of lineages that have experienced different amounts of affinity maturation: both naive lineages, and very mature ones, in the case of the bone marrow. Thus, it is hard to extract meaningful dynamical insight without knowledge of the underlying cell states. In the next section, we explore the underlying differentiation process of the individual B cell subtypes, which allows us to describe the dynamics of memory formation.

## Antibody-Secreting Cells Have at Least Four Distinct Phenotypes.

We initially labeled the cell types using celltypist, an algorithm for automatically assigning cell types to gene expression profiles (7). Our data revealed additional phenotypic variability within ASCs (see *SI Appendix*, Figs. S4, and S5 for an analogous analysis for memory B cells), consistent with a prevailing understanding that ASCs harbor substantial functional diversity (5, 14, 33). We detected ASCs expressing all isotypes. Interestingly, we also observed a substantial fraction of IGHD+ ASCs (*SI Appendix*, Fig. S4*E*); these ASCs were far more heavily hypermutated than ASCs of other isotypes, and represent a class-switched IgD population that arise in qualitatively different class-switch recombination processes and have specialized functions (34). ASCs formed four distinct clusters (ASC-1 through ASC-4) with notable differences in their gene expression (*SI Appendix*, Fig. S4 *A* and *D*). A substantial fraction of ASC-3 were proliferating and had a gene expression signature that closely resembles previous descriptions of plasmablasts (35) (*SI Appendix*, Fig. S4*D*). The other types of ASCs appeared nonproliferative and were commonly found outside the peripheral blood (*SI Appendix*, Fig. S4 *B* and *C*). In particular, ASC-1 are primarily located in the bone marrow and have the canonical markers of long-lived plasma cells (5, 36) (*SI Appendix*, Fig. S4 *B* and *D*). We noted the ASC-1 subset expressed high levels of FCGRT compared to other subsets of plasma cells, suggesting they may have specific regulatory mechanisms associated with local circulating antibody levels or autocrine signaling mechanisms related to secretion of their own antibody (*SI Appendix*, Fig. S4*D*).

## B Cells Differentiate at Constant Relative Rates Throughout the Germinal Center Reaction.

Similarly to the observations we made with respect to B cells located in different tissues, we find systematic differences in the hypermutation levels of different B cell types (Fig. 4*A*). However, within lineages, we cannot detect statistically significant differences in the level of hypermutation between related cells of different types, with the exception of naive B cells (Fig. 4*B* and *SI Appendix*, Figs. S6*A* and S7*A*, note the same does not apply to B cells of different isotypes; see *SI Appendix*, Figs. S6*B* and S7*B*). This surprising observation suggests that, during affinity maturation, the relative probabilities of differentiation into a memory B cell or an ASC remain constant. In other words, it argues against a temporal switch model in which ASCs arise preferentially later in the course of affinity maturation (17). Thus, as in the case of the tissue-level hypermutation differences, this represents an instance of Simpson’s paradox, where an apparent repertoire-wide correlation of hypermutation level with cell type is driven by differences in hypermutation levels between lineages with different cell type compositions.

To investigate this hypothesis further, we formulated the simplest possible model of differentiation during the germinal center reaction consistent with these observations. Specifically, we considered a model in which B cells in the germinal center have a constant probability of dying or exiting the germinal center as one of the B cell subtypes every time they re-experience selection in the light zone of the germinal center (i.e. each “germinal center cycle”); see Fig. 4*C*. In this model, the distribution of hypermutation levels for each of the B cell subtypes is exponential with a rate equal to the per-cycle event rate (death or differentiation; see *SI Appendix*, section F). This per-cycle rate is equal for cells of all types, and results in a uniform distribution of hypermutation levels among cells of different types in a lineage, as is observed in the data (Fig. 4*B*).

The repertoire-wide differences between numbers of hypermutations within a lineage suggest that there must be systematic differences in the differentiation rates of lineages with different cell type compositions. Differences between lineages could be driven either by intrinsic differences between the initial B cell receptors, intrinsic differences in the transcriptional states of the cells entering the germinal centers, or differences in the environment of particular germinal centers. This raises the question of whether such differences between lineages drive an apparent repertoire-wide change in germinal center exit rates as hypermutations accumulate.

To investigate this, we quantified the repertoire-wide effective germinal center exit rates for each of the cell types from the cell type hypermutation distributions (*SI Appendix*, section F.2). Remarkably, for cells that have accumulated over 10 hypermutations, these effective exit rates are constant. (Fig. 4 *A* and *D* and *SI Appendix*, Fig. S29). At lower hypermutation levels, all cell types deviate from their respective long-term effective exit rates by a similar multiplicative factor, which suggests there is an overall change in the rate at which long-term B cell memory is formed from weakly hypermutated B cells. However, because the fractional reduction in the exit rate is equal for the different cell types (Fig. 4*D*), the relative rates at which memory B cells and ASCs are created remain remarkably consistent throughout the germinal center reaction, with memory B cells being created in each germinal center cycle at an estimated rate that is roughly 40% higher than that of ASCs (*SI Appendix*, Table S3). Thus, we do not observe a repertoire-wide shift in the differentiation biases in the germinal center reaction.

However, the difference in the absolute effective exit rates explains the observation that ASCs tend to be more hypermutated than memory B cells: Since differentiation into an ASC occurs with a lower repertoire-wide probability than differentiation into memory B cells, we expect ASC creation to occur after on average more germinal center cycles (Fig. 4*E*). Finally, we compared these observations to the results of a previously published in vitro human B cell differentiation experiment (32). In that work, we observed that hypermutated B cells were intrinsically more likely to differentiate into ASCs than unhypermutated B cells when stimulated with the same T cell–dependent stimulus in vitro. We reanalyzed these data to quantify how the differentiation bias changes across hypermutation levels and found that the probability of differentiating into an ASC is uniform across hypermutation levels, if a B cell has accumulated order 10 hypermutations prior to in vitro differentiation (Fig. 4*F*). This experimental observation shows that no population-level changes in these intrinsic biases are observable in vitro across a range of hypermutation levels consistent with our measurements in human donors.

## Differentiation Outcomes of Related Cells Are Independent, Except For ASC-3s.

**Fig. 5.**
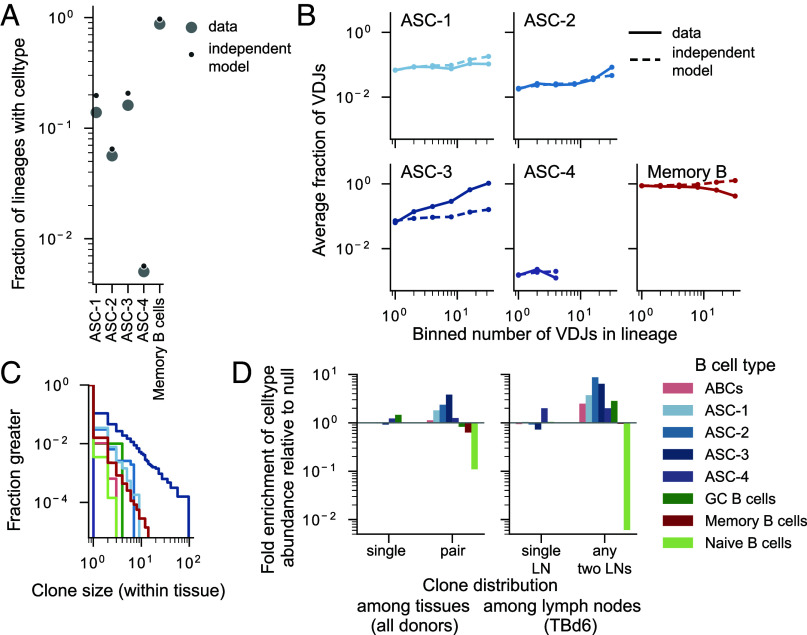
Distributions of cell types among cells belonging to the same lineage. (*A*) The total fraction of lineages with at least 2 unique VDJs that contained at least one cell of each type and (*B*) The average fraction of cells of a given type among lineages of a certain size. To construct the null expectations, we conditioned on the sampled phylogenies of all hypermutated cells with available transcriptomes and assumed independence between sampled cells. Points supported by fewer than 6 cells and fewer than 10 lineages have been omitted. (*C*) Clone size distributions of different B cell subsets. The clone size is the number of cells of each cell type that have an identical heavy chain nucleotide (VDJ) sequence and are found in the same tissue. Colors as in (*D*), which shows the changes in abundances of B cell subtypes as a function of the tissues their VDJ sequences were found in. As explained in *SI Appendix*, section E.2, VDJs are associated with a subtype label if they are found in a B cell of that subtype in any tissue. The null expectation is computed from the marginal subtype distributions across the tissues.

This view of differentiation in the germinal center implies that the cell type composition of a lineage can be predicted by the amount of hypermutation observed in the lineage. To verify this, we constructed a null probability distribution of B cell types as a function of the number of hypermutations observed in the templated IGHV gene from the data (Fig. 4 *E*, *Left*). We found that this model predicts both the probability that a lineage contains a cell in a certain state (Fig. 5*A*), as well as the total number of cells found in a certain state as a function of lineage size (Fig. 5*B*). Notably, this is not true for ASC-3s, which are found in far larger numbers in large lineages than expected. Thus, ASC-3s arise in a correlated fashion within certain lineages. In other words, there appears to be a global property of certain B cell lineages that may make them more prone to generating more ASC-3s.

Given the resemblance of ASC-3s to plasmablasts, which are thought to arise during active immune responses, this raises the question of whether ASC-3s preferentially arise in recently generated, ‘young’ lineages of B cells. To test this, we reasoned that, because human naive B cells are thought to have a half-life of only several weeks (37), lineages in which we observe an unhypermutated naive B cell must be younger on average. Memory B cells sampled in these lineages typically carried fewer hypermutations (*SI Appendix*, Fig. S8*A*), consistent with the interpretation that they are newly generated. However, not only did these “young” lineages not contain an abundance of ASC-3s, but ASC-3s accounted for a far higher proportion of cells in lineages that did not contain naive B cells (*SI Appendix*, Fig. S8*B*). This suggests that a global property other than age controls the abundance of ASC-3s in lineages. These findings are intriguing in light of a recent finding by Phad et al. (38), which showed that proliferative ASCs in the peripheral blood are often members of persistent lineages and are observable long after antigen encounter. Their observations likely pertain to the same ASC subtype, as ASC-3s account for almost all of the measured ASCs in the peripheral blood (*SI Appendix*, Fig. S4*B*).

## Expanded B Cell Clones Are Often Shared Across Tissues.

Finally, we noticed that about 1 in 100 of all unique nucleotide VDJ sequences were identical in many individual B cells within the same donor, suggestive of extensive clonal expansion without hypermutation (*SI Appendix*, Fig. S10*A*). This observation cannot be explained by the repeated generation of “public” B cell clones, which arise at rates that are several orders of magnitude lower (*SI Appendix*, section D and Fig. S9). Though evidence of clonal expansion can be found among cells of all types, it is particularly prominent among ASCs, especially ASC-3s (Fig. 5*C*).

**Fig. 6.**
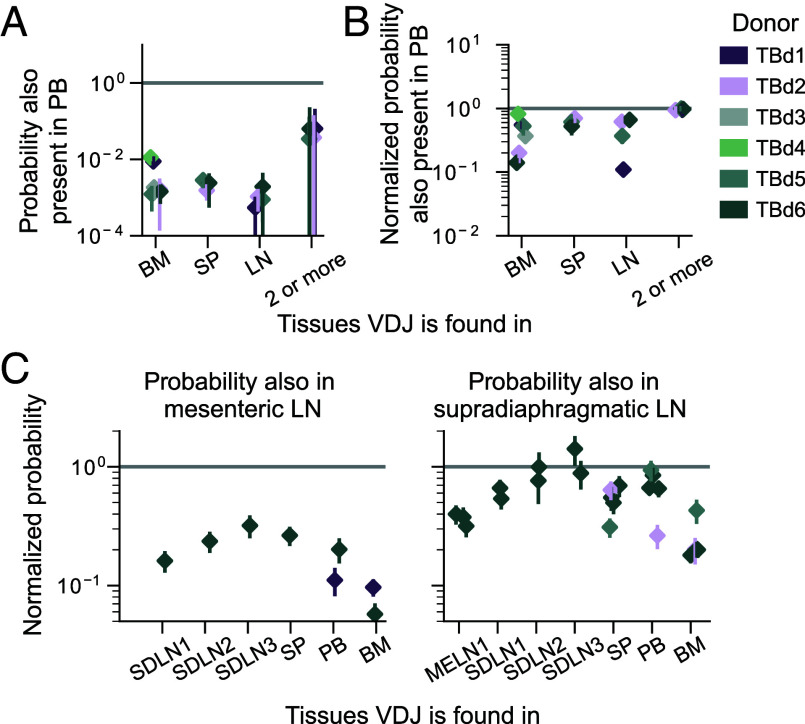
Localization and identities of shared B cell clones. (*A*) Probability that a B cell clone is found in the peripheral blood, given its VDJ has been sampled in a different tissue. In (*B*), we normalize the probability of appearance in the blood by the probability that the same VDJ was encountered in an independent sample of the tissue indicated on the *x*-axis (see *SI Appendix*, section E for details). This effectively conditions on expansion in the original tissue. To make symmetric comparisons between tissues sampled at different depths, all samples were downsampled to 3,000 unique VDJs per independently generated 10X emulsion from a cell suspension, and smaller samples were dropped from the analysis. Individual points represent averages of 100 independent samples of the entire dataset, and error bars denote the SE obtained from independent 10X runs of the same tissue, when available. (*C*) Probability that a B cell clone is found in a specific lymph node, conditional on it being expanded in another lymph node or nonlymph node tissue. MELN—mesenteric lymph node; SDLN—supradiaphragmatic lymph node.

Our analysis of the tissue localization statistics of lineages showed that, when lineages reach a threshold size, they have an order one probability of being found in multiple tissues. We found that expanded clones had a similar pattern. We quantified how often expanded clones were restricted to a single tissue. Once we accounted for the variability in the number of VDJs detected in each sample, we found that, across donors, all pairs of tissues shared similar fractions of VDJs (Fig. 6*A* and *SI Appendix*, Fig. S10*B*). Since even replicates of the same tissue have limited overlap in the context of limited sampling, we derived an empirical estimate of the sampling probability of an expanded VDJ using independent samples of the same tissue (*SI Appendix*, section E). We then normalized the measured probability that a VDJ sequence was present in a second tissue by this empirical probability of sampling a present sequence. Surprisingly, VDJs expanded in one tissue are found in other tissues with normalized probabilities often exceeding 0.1 (Fig. 6*B* and *SI Appendix*, Fig. S10*C*), and VDJs found in at least two tissues appear in a third, or fourth tissue with normalized probabilities of order 1. These observations suggest that expanded clones often achieve universal distribution throughout the sampled organs.

Earlier work revealed distinct networks of B cells associated with different anatomical locations (26). We investigated whether there was additional observable structure among lymph nodes from different anatomical locations. Consistent with these earlier findings, mesenteric lymph nodes shared notably fewer clones with all other tissues than supradiaphragmatic lymph nodes (Fig. 6*C* and *SI Appendix*, Fig. S10*D*), and distinct supradiaphragmatic lymph nodes shared clones among themselves as often as they did with other tissues (Fig. 6*C* and *SI Appendix*, Fig. S10*C*).

Multitissue clones are predominantly ASCs, and in particular ASC-3s (Fig. 5*D*). Still, we detected many shared memory B cell clones between tissues. Shared memory B cells had distinctive gene expression features compared to memory B cells which were not shared between tissues. We found shared memory B cell clones had a gene expression program distinguished by MAST4 and a nonswitched phenotype (*SI Appendix*, Fig. S11 *A* and *B* and section E.3). Interestingly, the gene expression signature we identified using clones detected in the Spleen and Lymph Node seemed to universally enrich for shared memory B cell gene expression among any pair of tissues containing a secondary lymphoid organ (*SI Appendix*, Fig. S11 *B* and *C*).

## Discussion

In this work, we measured the VDJs and transcriptomes of B cells sampled from a number of organs from six human donors. This allowed us to investigate the statistics of B cell evolution, migration, and differentiation. We assess the amount of overlap in antibodies produced by B cells residing in different organs and find that related B cells tend to colocalize in the same lymphatic tissue. This effect was particularly prominent in the bone marrow, which contained the greatest enrichment of large, private B cell lineages not observed in the other lymphatic organs. Thus, there is substantial spatial structure in the B cell immune system that should inform the interpretation of peripheral blood draws for the purpose of B cell repertoire monitoring.

The colocalization of related B cells suggests that many of them acquire coherent localization signals during their generation. This coheres with observations made in ref. 39 showing that qualities of B cell memory recall depend on the relative anatomical locations of the prime and boost during vaccination. However, a subset of ASCs (here labeled ASC-3) with properties similar to plasmablasts depart from this pattern. These cells collectively account for about 1 in 100 of all of the sampled VDJs and achieve universal distribution among the sampled organs. They represent the predominant mode of clone sharing between all sampled tissues, including in distinct pairs of anatomically colocated lymph nodes.

Though we have not sampled any lymph nodes with evidence of ongoing affinity maturation, we find that anatomically similar lymph nodes share memory B cell clones at rates no higher than other pairs of tissues. These findings have interesting implications for germinal center dynamics, especially in light of work showing that antibody evolution is shaped by secreted antibodies (40–42). They suggest the evolutionary outcomes of germinal centers may be modulated through the effect of sharing of recently created, cycling ASCs from distant areas of the body.

Our analysis of long-term B cell memory shows that related B cells that have undergone different amounts of affinity maturation (as evidenced by their hypermutation levels) have remarkably uniformly distributed probabilities of localization and differentiation. It is possible that other, transient populations of differentiated B cells are created during immune response and deviate from these patterns, and studying their dynamics in light of our results is an interesting avenue for future work. The incidental inclusion of the donor that had undergone COVID-19 booster vaccination within 30 d of our measurement suggests that there may indeed be differences in the transient populations that arise during immune response, as evidenced by the presence of atypically large lineages in this donor and an excess of sharing of VDJs between the bone marrow and the blood. Interestingly, this excess of shared VDJs is not observed in the sampled mesenteric lymph node (*SI Appendix*, Fig. S3).

In contrast with previous work that has suggested a temporal switch in which germinal centers become biased toward ASC production in the later phases of affinity maturation (17), our data are consistent with a model of B cell differentiation during affinity maturation in which differentiated subtypes of cells arise at a constant relative rate at all but the weakest hypermutation levels (>3%). Moreover, more than 80% of differentiated B cells exit the germinal center at a point in affinity maturation when differentiation rates have stabilized (*SI Appendix*, Fig. S30). Our observations are consistent with recent studies conducted in mice, which showed that long-lived ASCs continuously populate the bone marrow during an immune response (19, 43). Thus, we suggest long-lived ASCs are created and disperse to long-term niches early during the human immune response as well, albeit more rarely than comparable memory B cells.

Our statistical inferences from the human antibody repertoire cohere with recent single pathogen studies in mice (44, 45) that raise questions about whether antibody affinity is a determinant of B cell differentiation outcomes (15). If affinity does affect differentiation, it may be that high-affinity antibodies often arise very early during affinity maturation, or that the affinity threshold governing differentiation potentials becomes more stringent as the germinal center reaction proceeds. These two pictures have different implications for both our understanding of the dynamics of affinity maturation as well as practical applications, including in vitro antibody design and vaccine design. Future work that combines single-cell antibody repertoire sequencing with systematic measurement of the binding affinities along lineages would distinguish between these two pictures.

## Materials and Methods

Donated organs and tissues were procured at hospital locations in the Northern California region through collaboration with, Donor Network West (DNW, San Ramon, CA, USA). The research protocol was approved by the DNW’s internal ethics committee (Research project STAN-19-104) and the medical advisory board, as well as by the Institutional Review Board at Stanford University which determined that this project does not meet the definition of human subject research as defined in federal regulations 45 CFR 46.102 or 21 CFR 50.3. Fresh, whole, and nontransplantable organs were obtained from surgery and transported on ice by courier. Upon receipt, tissues were dissociated into single-cell suspensions using methods developed by the Tabula Sapiens Consortium (30). Some cell suspensions were processed immediately (fresh), and most were frozen and stored in liquid nitrogen. With the exception of the lymph nodes, all samples were enriched for the B cell lineage by selective depletion of non-B lineages by immunodepletion with magnetic beads. We used 10X 5^′^ immune profiling to encapsulate cells and barcode mRNA for single-cell library prep. Cells were loaded at a target of 20,000 cells per lane for gene expression samples and 80,000 cells per lane for VDJ-only profiling. We used Cellranger 7.0.1 to generate cell by gene count tables and VDJ assemblies followed by custom pipelines to process the gene expression data and assemblies. All cell types were annotated manually using input from both the gene expression data and the VDJ sequencing data. To promote reproducibility, we used the Snakemake workflow manager (46). Detailed descriptions of all experimental protocols and analyses are provided in *SI Appendix*.

## Supplementary Material

Appendix 01 (PDF)

## Data Availability

Raw sequencing reads have been deposited in the NCBI BioProject database with identifier PRJNA1203354 (47). Associated metadata, as well as the source code for the raw data processing pipeline, downstream analyses, and figure generation, are available on GitHub at https://github.com/michael-swift/Bcell_memory_formation (48).
