# Supplementary material for: Long-term B cell memory emerges at uniform relative rates in the human immune response
Source: Proc Natl Acad Sci U S A. 2025 Feb 28;122(9):e2406474122. doi: 10.1073/pnas.2406474122 (PMC11892634; doi:10.1073/pnas.2406474122)
Supplement: Supplementary file 1 — Appendix 01 (PDF) [file pnas.2406474122.sapp.pdf]

PNAS

Supporting Information for

Long-term B cell memory emerges at uniform relative rates in the human immune response

Ivana Cvijović*, Michael Swift*, Stephen R. Quake

Stephen R. Quake
E-mail: steve@quake-lab.org

This PDF file includes:

Supporting text
Figs. S1 to S30
Tables S1 to S3
SI References

Supporting Information Text Contents

A Experimental Methods . . . 3
A.1 Tissue processing . . . 3
A.2 Transcriptome and VDJ sequencing . . . 4
B Gene expression analysis . . . 4
B.1 Gene expression data preprocessing . . . 5
B.2 Doublet detection and automatic annotation . . . 5
B.3 Cell cycle assignment . . . 5
B.4 Batch integration . . . 5
B.5 Biases in cell type counting . . . 6
B.6 Fine-grained annotation of B cell subtypes . . . 6
C VDJ sequence analysis and cell calling . . . 6
C.1 VDJ sequence preprocessing . . . 6
C.2 Cell calling, V-gene tree construction, and detection and removal of cross-contaminants . . . 7
C.3 Light chains . . . 8
C.4 Detection and removal of cross-contaminants . . . 8
C.5 Transcriptome-informed analysis and annotation of ambient transcripts . . . 9
D Analysis of heavy chain sharing between donors . . . 10
E VDJ and lineage sharing analysis . . . 11
E.1 VDJ sharing between tissues and subanatomical regions . . . 11
E.2 Enrichment of cell types among shared VDJs . . . 12
E.3 Gene expression associated with shared VDJs . . . 13
E.4 Lineage sharing between tissues . . . 13
F B cell differentiation during affinity maturation . . . 13
F.1 A constant rate model of B cell differentiation . . . 13
F.2 Empirical quantification of repertoire-wide exit rates for different cell types . . . 14

List of Figures

S1 B cell type distribution across donors and tissues. . . . 16
S2 Cycling phenotype across tissues and cell types. . . . 17
S3 Distributions of cell types among cells belonging to the same lineage. . . . 18
S4 Characterization of ASC transcriptomes and VDJs. . . . 19
S5 Characterization of in memory B cell transcriptomes and VDJs. . . . 20
S6 Distributions of cell types and isotypes among cells belonging to the same lineage. . . . 21
S7 Distributions of cell types and isotypes among cells belonging to the same lineage. . . . 22
S8 Correlations in cell types of cells belonging to the same lineage. . . . 23
S9 Characteristics of VDJ sequences found in multiple donors. . . . 24
S10 Clonal expansion and hypermutation distributions across tissues and localization statistics of shared B cell clones. 25
S11 Gene expression signatures of shared memory B cells. . . . 26
S12 Sample depletion by antibody-secreting cells. . . . 27
S13 Cell cycle annotation. . . . 28
S14 Silhouette scores upon batch correction/integration. . . . 29
S15 Biases in relative abundances of B cell types during sampling. . . . 30
S16 Biases in cell subsets due to the freeze-thaw procedure . . . 31
S17 Memory B cell phenotypes. . . . 32
S18 Distribution of putative cross-contaminating heavy chains. . . . 33
S19 Distribution of putative cross-contaminating heavy chains among the pair of samples between which they are shared. . . . 34
S20 Heavy chain UMI abundance among shared cell barcode-VDJs and private cell barcode-VDJs among samples involved in any shared cell barcode-VDJ events. . . . 35
S21 Abundance of ambient VDJs. . . . 36
S22 Distribution of ambient VDJs among droplets . . . 37
S23 Distribution of ASC-associated VDJs among droplets . . . 38
S24 Presence of rare class-switched ambient contaminants within droplets with Naive B cell gene expression. . . . 39
S25 Misidentification of ambient VDJs with GEX signatures . . . 40
S26 Rates of VDJ multiplicity in droplets . . . 41
S27 Sharing of lineages between pairs of tissues. . . . 42
S28 Schematic illustrating a model of a memoryless differentiation process within an active germinal center. . . . 43
S29 Scaled logarithm of the complementary CDF of the hypermutation number distribution. . . . 44
S30 Distribution of hypermutation numbers in all cells found in lineages with at least 3 sampled unique VDJs . . . 45

List of Tables

S1 List of all tissues used in this study and their anatomical locations. 46
S2 Enumeration of all sequencing libraries analyzed in this study. 48
S3 Inferred per-cycle exit rates for the model implied in **Fig. S28**. 49

Supporting Information Text

A. Experimental Methods.

A.1. Tissue processing.

Tissue procurement. Donated organs and tissues were procured at various hospital locations in the Northern California region through collaboration with Donor Network West (DNW, San Ramon, CA, USA). DNW is a not-for-profit, federally mandated organ procurement organization (OPO) for Northern California. Recovery of non-transplantable organ and tissue was considered for research studies only after obtaining records of first-person authorization (i.e., donor's consent during their DMV registrations) and/or consent from the family members of the donor. Tissues were processed consistently across all donors. Each tissue was collected and transported on ice, as quickly as possible to preserve cell viability. A private courier service was used to keep the time between organ procurement and initial tissue preparation to less than one hour. Single cell suspensions from each organ were prepared as described below.

Background of donors. The brief medical history for all donors is outlined in **Table 1** in the Main Text. To be included in our study, donors had to have no history of immune disease or cancer, and no evidence of active infection or pregnancy. Per DNW Management Guidelines, all donors received a number of drugs while on a respirator prior to cross clamp for organ removal. During this time, all donors received antibiotics. Zosyn was the most common, but Diflucan and Vancomycin were given to some donors. Based on culture results, drug allergies or medication availability, other antibiotics were administered to some donors. Donors also received anti-coagulants, anti-inflammatories, diuretics, and drugs to maintain blood pressure. Most commonly these were heparin, Solu-Medrol, Levophed, and Lasix.

Tissue processing procedure. All 6 donors were processed with standard protocols previously described in Ref. (1). We reiterate them below for completeness.

Blood We mixed the full amount of blood from the organ donors (between 5 and 40mL) with an equal volume of PBS plus 2% BSA. 15 mL of Density Gradient (Ficoll Histopaque-1119) were added to empty 50-mL Falcon tubes. Up 25 mL of blood/buffer mixture were added to each Ficoll-filled Falcon tube, tilting the tube and pipetting on its side to prevent the blood from mixing with the Ficoll. Tubes were centrifuged at 400g for 30 minutes at room temperature, with the centrifuge brakes off. After centrifugation, the tubes were inspected to check that all layers were well separated. Starting from the bottom, the following layers were identified: erythrocytes, Ficoll solution, buffy coat with cells (white color), and plasma. The buffy coats were gently removed from each tube and transferred into a new 50mL Falcon tube. 30mL of cold (4°C) PBS + 2% FBS were added to each buffy coat. After pelleting the cells in the buffy coat, we washed them twice in cold PBS + 2% FBS, then performed ACK Erythrocyte Cell (ThermoFisher) lysis for 5 minutes. After the ACK lysis step, cells were washed with 10mL of ice-cold PBS and spun down at 4°C, 500g for 10 minutes. Cells were then resuspended in buffer and enumerated using a hemocytometer.

Bone Marrow The vertebral bodies (VB) were wrapped in a saline-soaked cloth and shipped to Stanford University on ice. Upon arrival, the VBs were cleaned of connective tissue and fat using sterilized wood chisels. VBs were then cut into $\sim 2\text{cm}^3$ pieces using bone cutting forceps and rongeurs. The bone marrow pieces were transferred into a plastic container, to which 100µL of RPMI + 10% FBS was added. The container was mechanically tumbled for 30 minutes at room temperature. At this point the buffer was turbid and cellular. This was then passed through a 100 µmstrainer into 50mL falcon tubes which caught bone chips and other smaller debris. Multiple strainers were often used due to clogging. After straining, the cells were centrifuged and pelleted at 330g for 5 minutes at 4°C . Cells were then washed twice with PBS + 2% FBS andresuspended in PBS + 2% FBS. BMMNCs were then isolated using Ficoll by layering 35mL of cell suspension on 15mL of Ficoll density gradient medium. Cells were centifuged at 445g for 35 minutes at 20°C in a swinging bucket rotor without braking. Mononuclear cells were transferred into a new 50mL Falcon tube. 30mL of cold (4°C) PBS + 2% FBS were added to each buffy coat. After pelleting the cells in the buffy coat, we washed them twice in cold PBS + 2% FBS, then performed ACK Erythrocyte Cell lysis for 5 minutes. Cells were then centrifuged for 5 minutes at 330g at 4°C without brakes. Cells were then resuspended in PBS + 2% FBS and enumerated using a hemocytometer.

Spleen The spleen tissue was placed in a petri dish and minced with sterile surgical scissors and scalpels. The minced tissue was transferred to a 50mL conical tube with 5mL of digestion media, which was freshly prepared (0.8 mg/mL Collagenase IV (Worthington) and 0.05 mg/mL DNase I (Roche) in RPMI with 10% FBS). The tissue was further minced with scissors inside the 50mL tube. The petri dish containing the tissue was washed with 5mL of digestion media, and everything was transferred to the 50mL tube. The tissue was digested in a shaker at 37°C, 200 rpm for 30 minutes. The sample was vortexed every ten minutes to re-disperse the tissue. After digestion, we pipetted the mixture vigorously to evaluate digestion and to further mechanically digest the tissue as much as possible. The tissue solution was diluted in RoboSep buffer (Stemcell Technologies) and then passed through a 100µm cell strainer into a 50mL tube. A plunger from a 10mL syringe was used to

mash the remaining tissue in the strainer. The cell suspension was spun at 4°C, 450g for 10 minutes and washed twice in RoboSep buffer. Finally, we performed ACK Erythrocyte Cell lysis for 5 minutes, after which cells were washed with 10mL of ice-cold PBS and spun down at 4°C, 500g for 10 minutes, then enumerated with a hemocytometer.

Lymph Nodes Individual lymph nodes were collected in a p100 petri dish and the surrounding edges were cleaned to extract lymph nodes from surrounding fat, using sterile surgical scalpels and scissors. Once the vast majority of fat was removed, the lymph nodes were placed in a 5mL polypropylene tube and minced with sharp scissors. Digestion media was prepared with 0.8 mg/mL Collagenase IV (Worthington) and 0.05 mg/mL DNase I (Roche) in RPMI plus 10% FBS. Up to 5mL of digestion media was added to the 5mL polypropylene tube, which was then placed in a shaker to digest at 37°C, 200 rpm for 20 minutes. At the end of the 20 minutes, the cell suspension was pipetted vigorously up and down 10 times to evaluate digestion and to further mechanically digest the tissue as much as possible. The tissue solution was diluted in RoboSep buffer and then passed through a 100µm cell strainer into a 15mL tube. A plunger from a 10mL syringe was used to mash the remaining tissue in the strainer. Cells we spun down and resuspended in RoboSep buffer. After an optional ACK lysis step (based on whether the pellet looked red), cells were spun down at 4°C, 450g for 10 minutes, resuspended in 5mL of buffer and enumerated with a hemocytometer.

Cell freezing and thawing For freezing, cells were spun down and resuspended with around 200μL of RoboSep buffer. Ice-cold Cryostor CS10 buffer was added to the cells to attain a density between 10 and 40 million cells per mL. The cell suspensions were transferred to cryovials, and frozen using a standard slow rate-controlled cooling protocol (approximately -1°C/minute) in CoolCells. in a -80°C freezer. After a few hours cells were moved to long-term storage in liquid nitrogen vapor. For thawing, we followed the 10X Genomics thawing protocols, which appear to differ from typical protocols only in that they add complete media, RPMI + 10% FBS (or similar), at a slower rate. Initially, we experienced catastrophic amounts of cell aggregation during thawing, in particular in bone marrow cells, which was mitigated by using 50U/mL of Benzonase (Sigma-Aldrich) in the thawing media.

B cell purification and loading on Chromium or Chromium X Cell suspensions were counted and resuspended at target densities of 5×10^7 cells per mL. B cell purification was performed according to manufacturer instructions with the Stem Cell EasySep Human Pan-B Cell Enrichment kit (Stemcell Technologies). After enrichment, cells were resuspended in RoboSep Buffer and counted. We loaded cell suspensions on the Chromium or Chromium X (10X Genomics) instruments according to manufacturers instructions outlined in User Guide version CG000510 Rev B for the Chromium and version CG000512 Rev B for the Chromium X, except when performing VDJ only profiling. We targeted 20,000 recovered cells per lane when measuring gene expression and VDJ profiling and 80,000 recovered cells per lane when performing VDJ only profiling.

A.2. Transcriptome and VDJ sequencing. Gene expression libraries were generated following the 10X User Guide CG000510 Rev B for non-HT libraries and and User Guide CG000512 Rev B in the case of HT libraries. VDJ amplicons were amplified and enriched by a nested PCR using the same primer sequences as quoted by the 10X manual, but in custom PCR mixes, containing KAPA 2X HotStart (Roche), the 10X forward VDJ amplification primer at a final concentration of 1µM, and the pool of 10X isotype-specific reverse primers at a final concentration 0.5µM each and in a total volume of 50µL. Both the inner and outer target enrichment PCRs were performed for 8 cycles, annealing at 67°C. We then proceeded to make libraries from these amplicons according to the 10X User Guides, using the versions noted above. Individual libraries were dual-indexed and sequenced on Illumina NextSeq 2000, or Illumina NovaSeq S2 or S4 flowcells. We targeted a sequencing depth of 5000 reads per cell for VDJ amplicons and 40 000 reads per cell for the gene expression libraries.

We noted that antibody secreting cells express on average multiple orders of magnitude more antibody transcripts than naive or memory B cells, which is unsurprising given the majority of detectable RNA molecules in antibody secreting cells are derived from the IGH and IGK/L loci(2). Specifically, whereas the reconstructed IGH amplicons for most B cells in our study were supported by fewer than 20 IGH UMIs (**Fig. S12**, bottom panel), 1-10% of cells in each sample were supported by thousands or even tens of thousands of UMIs. As a result, the majority of captured transcripts, and thus the majority of captured reads, is associated with these cells. This results in the preferential detection of antibodies of ASC origin in our sequencing libraries, and the corresponding loss of VDJ transcripts associated with memory or naive B cells, which have vastly lower expression.

To understand whether, in principle, sequencing more deeply would allow us to detect additional antibody transcripts from naive and memory B cells we examined what fraction of UMIs were available to the typical cell in our samples (**Fig. S12**, top panel). We found that in all samples the fraction of total molecules sequenced from ASCs was over 90%. In certain samples, a small number of ASCs accounted for almost 999 in 1000 reads, with 99 in 100 being the more typical rate. Thus, even when targeting 5000 reads per cell, this effectively allocated no more than 5 to 50 reads to the median memory or naive B cell, causing them to frequently drop out from our libraries. As a result, we concluded that full recovery of our naive and memory B cell transcripts would require us to target about 500,000 reads per cell in the VDJ amplicon libraries, a depth currently practically infeasible given the scale of our study and the cost of sequencing.

We note that this problem is fundamental to ASC presence in a mixed library, and arises in all circumstances in which these cells are not separated from memory and naive B cells prior to lysis. As such, it persists when sequencing antibody transcripts in bulk. Thus, we recommend that researchers physically separate cells with ASC phenotypes from memory and naive B cells, prior to lysis, to ensure the balanced recovery of transcripts from each of these subsets.

B. Gene expression analysis.

B.1. Gene expression data preprocessing. We used `cellranger v7.0.1` to align reads and count UMIs from the raw FASTQ gene expression data. Reads were aligned to the GRCh38 human reference genome. The unfiltered outputs of `cellranger` were used as inputs to `cellbender`(3), which develops a per-sample model of ambient RNA and produces a count matrix that has removed putative ambient RNA. While we used these count matrices for orientation in exploratory analysis, all cell type annotation and gene expression analyses were ultimately based on the raw UMI counts. Cell barcodes with fewer than 1000 UMIs or with fewer than 500 genes detected were excluded from downstream analysis. To facilitate doublet-detection, detect batch effects, and gain additional statistical power for cell type identification, we added the entire dataset from Ref. (4) to our pipeline before training scVI models. The dataset was downloaded from the Ref. (5). Pan Immune Dataset cells were not included in any of the analyses, statistics and visualizations presented in the manuscript with the exception of **Fig. S14**.

B.2. Doublet detection and automatic annotation. We performed doublet detection on a per-sample basis using `scrublet` (6). We noticed the algorithm likely had a high proportion of negatives, given the inferred doublet rates were often far lower than the likely doublet rate given our loading densities ($\sim 10\%$). Thus, we employed two "faction-voting" methods and one gene-based heuristic to flag cells as doublet-associated, regardless of their doublet score.

First, we flagged cells if their gene expression signature is similar to that of other cells with high doublet scores. We labeled these cells "doublet-associated". Doublet-association is defined as at least 1 in 10 of the cells in a given Leiden cluster are called doublets by the Scrublet algorithm, all cells in that cluster are flagged as doublet-associated.

Second, we flagged cells based on `celltypist` labels for their cluster. Using the "Immune_All_High" model, we predicted broad cell type labels for each cell (e.g. "B cell", "Macrophages"). We then clustered all cells using (`sc.tl.leiden(resolution = 3)`). Clusters with mixed membership (i.e. with a minority member fraction > 0.1) were also flagged as doublet-associated by cell voting. While this heuristic generally performed very well, cycling cells such as plasmablasts were often flagged as doublet-associated. We believe this is due to their strong cycling signature causing them to cluster with cycling cells of other immune lineages. Thus, we exempted cycling cells from this flag. Finally, using gene expression signatures of observed non-B cell contaminants, we created contaminant gene scores `sc.tl.score_genes()` using MPO, AZU1, ELANE, and S100A8 as the Myeloid score, and CD3E, CD3D, and CD247 as the T cell score. For subsequent integration with VDJ contig data, we labeled cells as probable high-quality single B cells if:

- `doublet_score` is less than 0.01,
- `t_cell_score` is less than 0,
- `myeloid_score` is less than 0,
- `celltypist` identifies the cell as a B cell
- `celltypist` confidence score is > 0.95

Only cells labeled as high-quality single B cells were used in analyses of B cell transcriptomes. Cells labeled as high-quality single non-B cells were used to further quantify rates of ambient VDJ transcript encapsulation.

B.3. Cell cycle assignment. We used MKI67 as a marker of cell cycle status. However, we noted that in cell types we often did not detect MKI67, even in plasmablasts which are thought to be cycling (**Fig. S13**, upper left). This is likely due to technical limitations in sensitivity via droplet-based RNA sequencing. Thus, we took advantage of the co-variation between cell cycle genes to generate a more sensitive cell-cycle classification. First we calculated the correlation coefficient of all genes with MKI67. The distribution of the correlation values had a long tail of hundreds of genes with high (> 0.5) correlations. Manual inspection of the available evidence for the functions of these genes showed they were mostly involved in the G2/S phases cell cycle. We then used the top 30 most correlated genes to as a set create a cell cycle score using `sc.tl.score_genes()`. This score separated cell types known to be cycling from those that were not with better sensitivity and specificity (**Fig. S13**, upper right). We validated this score on a dataset of cycling B cells from Ref. (7), which we used to derive a threshold for calling cycling B cells (**Fig. S13**, bottom panels).

B.4. Batch integration. Technical or nuisance factors in our dataset may exist because we integrate data from multiple donors, whose donated tissue was processed on different days in subtly different ways (i.e. fresh vs. thawed from frozen), using subtly different encapsulation (i.e. the 10X Chromium vs. the 10X Chromium X). To investigate whether these clearly identifiable technical variables had systematic effects on the salient statistics for our analyses, such as the relative abundances of different cell types (i.e. whether freezing might selectively kill a certain cell type), we prepared replicates of peripheral blood and bone marrow samples from donor TBd3 using two different technical approaches. We discuss these findings in detail in the following section (**Section B.5**).

There can be a myriad of other more subtle or hidden technical factors that would affect gene expression measurements. Thus, for more granular labeling of cell types using unbiased clustering, it is important to be able to regress out transcriptome level technical effects while preserving biological variability. To this end, we used scVI (8) to build a model of gene expression for all cells in the dataset based on their raw UMI counts. The motivation for using scVI was that it has been shown to perform complex batch correction and differential expression tasks better than other methods such as BBKNN (9), at the expense of increased computational resources taken to train the models. (10). To train the scVI model we used the default `scvi.model.SCVI` parameters except we changed the number of latent dimensions to 30 and the number of layers to 2. With

the exception of the UMAP in **Fig. S4a** where the cell cycle score is used as a continuous covariate, all UMAPs in this publication were derived from the latent space of the scVI model (“X_{scVI}”) and trained jointly on the Tissue Immune Cell Atlas and the data generated here using the combination of “donor" and “tissue" as a batch key. Our scVI model compared favorably to other methods batch integration. For example, if we labeled the transcriptomes of every cell using `celltypist`, a well-regularized model that should be insensitive to batch effects, and then took these labels as a biological ground-truth, Leiden clustering of cells in the scVI latent space compared favorably to other approaches (PCA or BBKNN), as seen by higher average silhouette scores (**Fig. S14**).

B.5. Biases in cell type counting. Our B cell enrichment was performed via negative selection on all samples except those from the lymph nodes (in which B cells account for more than 30% of all cells even in the absence of enrichment) and two TBd5 peripheral blood samples, which we did not enrich because we had a very small amount of starting material for this donor and tissue (see **Table S2** for information about the specific sample identifiers this affected).

While enriching for B cells by negative selection afforded an essentially unbiased look at the relative abundances of different B cells, the enrichment product was nevertheless relatively impure, particularly in the bone marrow samples (**Fig. S15**, left panel). We noted that many of the contaminant cell types were progenitor hematopoietic cells ($\sim 17\%$) which may have escaped negative selection because of lower expression of lineage markers targeted by the selection antibody cocktail.

When we merged VDJ data with gene expression data on a per cell basis, we noted that many high quality B cell transcriptomes did not have a VDJ associated with them. This appears to be due to dropout of VDJ transcripts from B cells that have low antibody gene expression, such as Memory B cells (see also **Fig. S25** and the discussion in **Section A.2**). Thus, the set of VDJ transcripts that we captured is inherently biased towards antibody-secreting cells (see **Fig. S15**, right panel). As discussed in **Section A.2**, we anticipate that this bias is present in all unsorted B cell VDJ sequencing. We design all analyses that follow in such a way that they are minimally sensitive to this bias.

Finally, we investigated whether cryopreservation of samples leads to biases in B cell subtype composition, i.e. whether different subsets of B cells survive the freeze-thaw at different rates. To assess this, we prepared replicates of the peripheral blood and bone marrow cell suspensions from donor TBd3, the first immediately after receipt, the second after cryopreservation and thawing. As can be seen in **Fig. S16**, ASC-3s appear most prone to loss during the freeze-thaw procedure with an at least 3-fold survival deficit compared to other B cell subsets, followed by ASC-2s, with no meaningful changes to the abundances of other cell types (for details of the fine-grained annotation of the mentioned cell types, see **Section B.6**).

B.6. Fine-grained annotation of B cell subtypes. High-quality single B cells (i.e. cells that were not doublets, non-B cells, or have transcriptomes of dubious quality) were separated into Memory, Antibody Secreting Cell, and Naive types using the `Immune_All_Low` model and analyzed separately. While we explored a variety ways to analyze these gene expression profiles, ultimately we used the scVI representation to construct nearest-neighbors graphs (`sc.pp.neighbors`), which were clustered using the Leiden algorithm at resolutions between 0.5 and 1 (`sc.pp.leiden`). For ASCs, used an scVI representation with MKI67, RRM2, and TK1 supplied as continuous covariate keys to remove the contribution of the cell cycle. The resulting representation collapsed the distinction in the nearest-neighbors graphs between a particular cluster of Plasmablasts and Plasma cells (ASC-3) (**Fig. S4a**).

For memory B cells, we noted two major axes of gene expression variation beyond what is described by `celltypist` (**Fig. S5**). The first was explainable by class-switch status and the second axis was defined by CR2 and CR1 expression (**Fig. S17a**). Thus we used these genes to calculate a Complement Receptor score (`sc.tl.score_genes(adata, gene_list = [‘CR2’, ‘CR1’])`) and defined cells with a positive score as CD21++ (**Fig. S17b**). We incorporated antibody constant region information from the VDJ sequencing data by classifying cells as being switched (SW) if their assembly was not IGHM or IGHD, and non-switched (NS) otherwise. These two simple heuristics captured most of the detectable variability in B cell subsets, and agreed well with Leiden clustering results (**Fig. S17c**).

C. VDJ sequence analysis and cell calling.

C.1. VDJ sequence preprocessing. We used `cellranger v7.0.1` to filter, trim and assemble VDJ reads into contigs. Our study design included both high-density loading of the VDJ -only samples and highly variable VDJ expression in certain tissues, and we found that both of these can lead to overly aggressive filtering by the default `cellranger` VDJ annotation and cell-calling algorithm, which is designed with a very low tolerance for doublets. Since the focus of our study prioritizes VDJ detection in VDJ -only samples over the default standard of removal of all probable doublet droplets, we designed a custom pipeline to replace the default `cellranger` VDJ algorithm for contig annotation and cell calling. This allowed us to include all high-quality VDJ sequences in the analysis, while employing more stringent filtering on samples with paired gene expression data, as described below.

We used `IgBLAST v1.17.0` to annotate all assembled contigs produced by `cellranger` and further filtered the `IgBLAST` output to retain only high quality, productive VDJ transcripts. Only contigs satisfying all of the following criteria were retained:

- `v_support` is less than $\exp(-60)$,
- `j_support` is less than $\exp(-10)$,
- V gene alignment is at least 160 nucleotides long,

- J gene alignment is at least 20 nucleotides long,
- VDJ sequence is productive,
- VDJ sequence contains a CDR3,
- VDJ sequence contains no ambiguous bases.

For all purposes except for the providing evidence for the construction of the personalized V gene databases (see below), we further removed sequences that did not contain the full-length V gene (`v_germline_start<=2`). We assigned these high quality VDJ sequences to lineages in a germline V gene reference-free way by performing single linkage clustering on sequences with the same same CDR3 length, and the same V gene family, and with the clustering condition requiring that the neither the fractional Hamming distance between the CDR3 nucleotide sequences nor the fractional Levenshtein distance between the templated regions exceeds 0.15.

We then constructed a personalized germline V gene database for each donor using `grmlin` (11), and re-annotated the contigs by aligning them to this personalized V gene database using `BLAST v2.7.1` with the following additional options:

```
-word_size 9 -dust no -penalty -1 -gapopen 3 -gapextend 2.
```

By comparing the germline allele assignments found by `grmlin` and the germline assignments obtained by aligning the V sequence to the full IMGT database, we noticed that, in certain donors, there still remained a small number of likely germline alleles that remained undetected by `grmlin`. These were V genes that were found in their unhypermutated form in several lineages and did not represent hypermutations of other V genes in the personalized V gene database. For each donor, we ranked such additional candidate germline genes by the distance to the most similar gene in the personalized database, and then by the number of lineages the unhypermutated version of the gene was associated with. Since each of these candidate germline genes can be associated with it's closest and, by construction, more highly expressed gene in the germline database, we further calculated the ratio, r, of lineages associated with each of these genes. We then iteratively added these candidate germline genes to a refined germline database if all of the following conditions were satisfied:

1. the gene is supported by at least 5 lineages, OR is at least 5 mutations away from the closest germline gene in the database, and
2. the ratio, r, exceeds $0.3/d_{\mathrm{nearest}}$, where d_{nearest} is the Levenshtein distance between these two genes.

C.2. Cell calling, V-gene tree construction, and detection and removal of cross-contaminants.

Cell calling. A significant challenge of interpreting droplet-based single-cell experiment VDJ sequencing data is distinguishing between true cells and ambient transcripts. Specifically, because the generation of droplets occurs after cells have been loaded into lysis buffer, the lysis of some cells occurs *before* they have been encapsulated in their individual droplets. As a result, a large number of transcripts are released into the ambient. Distinguishing ambient VDJ transcripts from those associated with encapsulated cells becomes challenging, especially in light of the enormous variability in VDJ expression between antibody-secreting cells and naive and memory B cells, which can differ by more than two orders of magnitude (see **Section C.5** below). As a result, in the absence of gene expression data, it is in principle very difficult to distinguish between ambient and cell-associated VDJ transcripts.

Since one of the main quantities of interest in our study are the patterns of co-variation in the presence of different VDJs in different tissues, we employ a permissive approach to calling VDJ "cells" from this data, that we make more stringent in samples for which we do have gene expression data. Our approach has been designed with both long-read sequencing data applications (e.g. via the Pacbio platform), as well as reconstructed contigs of enzymatically fragmented amplicons (which, due to throughput limitations in long-read sequencing at the time of writing, is the exclusive source we use here, reconstructed by `cellranger`, see **Section C.1** above). We begin by verifying that all contigs are either associated with a whitelisted 10X cell barcode, or within 1 nucleotide of a single whitelisted 10X barcode. We further discard all contigs supported by only a single UMI.

We then proceeded to assign these contigs to possible cells by iterating through all droplets and deriving a limited number of consensus contigs for each immunoglobulin locus (IGH, IGK, or IGL) using the approach described in the remainder of this section. In droplets that contained a unique VDJ per locus supported by multiple UMIs, we simply retained this contig, labeling it as a potential cell. However, as anticipated in droplet-based single cell experiments, a fraction of droplets in all samples contained contigs of the same chain that had multiple distinct VDJ sequences. These could either represent multiple encapsulated cells, ambient contaminants, or uncorrected reverse transcription, PCR or sequencing errors.

To account for uncorrected errors arising in the library generation process, we first attempted to error-correct these sequences by deriving consensus sequences for each group of VDJs in the droplet with highly similar sequences. We did this by first constructing a minimum spanning tree for the VDJs associated with each immunoglobulin chain. We examined the distribution of distances between adjacent sequences in the minimum spanning tree, and found that it typically had a very rapid decay at small distances, and a small number of edges connecting very distant VDJs . This distribution is consistent with a small number of distinct, true VDJs surrounded by rare variants arising through errors in the library generation or sequencing process.

Motivated by this observation, the minimum spanning tree was then cut by removing all edges longer than 10 nucleotides. Thus, in each droplet, for each chain, we then had a collection of graphs representing connected components of VDJ sequences which likely only differed due to library generation errors. We assigned a consensus sequence to each of these connected components via parsimony: we chose the VDJ sequence within the component that would require the smallest total number of mutated bases to have occurred during library preparation to explain the presence of closely-related variants.

Thus, we reduced the contigs associated with each droplet to a small number of consensus VDJ sequences that either represented multiple encapsulated cells, ambient contaminants, or a combination of the two types of species. We reasoned that ambient contaminants were likely to be supported with a smaller number of unique molecules than true cell-associated VDJs . Thus, in droplets in which there were multiple VDJ of this type, we only retained VDJ supported by at least 4 UMIs and that accounted for no fewer than 10% of all the UMIs associated with that droplet. All other VDJs were designated "ambient".

Finally, we assigned a consensus C-gene call to each non-ambient VDJ. Due to sequence similarity among the different heavy chain isotypes, we found that, in a small fraction of cases multiple IGHC genes were associated with the same VDJ sequence in a cell. In these cases, we assigned the VDJ sequence the C-gene supported with the largest number of UMIs when that number was at least twice the number of UMIs supporting the second-ranked C-gene, and otherwise labeled the isotype as ambiguous.

Construction of V-gene trees. To construct phylogenetic trees of non-ambient templated V sequences within a lineage, we used `MUSCLE v5.1` to first construct MSAs of all unique V nucleotide sequences within the lineage. In cases when the germline version of the gene was not present in the lineage (i.e. we did not sample a naive B cell from that lineage), we also added the germline version of the most common V gene call within that lineage to the MSA, which later allowed us to root the V gene phylogenies on this sequence. We used `fasttree v2.1.11` to infer approximate maximum-likelihood phylogenies from these nucleotide MSAs, using the generalized time-reversible nucleotide evolution model and default parameters.

C.3. Light chains. In principle, IGK and IGL chains can be used to improve confidence in cell and lineage calls and to enable finer resolution of the phylogenetic tree. In practice, the inclusion of light chain information for the purposes of identifying clonal recombination events does not substantially improve accuracy (12). Moreover, given their comparatively lower diversity and higher expression compared to heavy chains, we find that associations with cells are often substantially more difficult to distinguish from cross-contaminants or ambient species. As we detail in the next sections, such distinctions are possible for heavy chains, and so we base all of our VDJ analyses exclusively on the heavy chain sequences. However, in all samples we sequenced and bioinformatically processed the light chains, since the paired heavy and light chain sequences can be used to produce monoclonal antibodies for future experiments. Reads associated with light chains have been made publicly available alongside the reads associated with heavy chain VDJ sequences.

C.4. Detection and removal of cross-contaminants. Since the parallel preparation of several dozen libraries always carries the risk of cross-contamination, we endeavored to remove all potential cross-contaminating reads prior to downstream analysis. To detect cross-contamination events, we exploited the enormous diversity of both heavy chain VDJ nucleotide sequences and of 10X cell barcodes. We reasoned that, though we expect that multiple samples may contain either the same VDJ molecule or the same cell barcode, it is very unlikely that a VDJ biologically shared between two samples is also associated with the same cell barcode. Specifically, given that we typically observe on the order of 100 shared VDJs even in replicates of the same tissue, and that there are about 737,280 possible cell barcodes by 10X design, the probability that there are any accidental collisions between any given pair of samples is on the order of 6×10^{-3}. Note that this still means that rare collisions are expected to occur in donors for which we have many samples of the same tissue, but that these collisions are expected to be rare, and that their removal should not significantly affect our estimates of rates at which sharing between tissues occurs.

Of the 1,160,896 unique cell barcode-VDJs in our dataset, we found 348 that were shared between multiple libraries (see **Fig. S18**). In all instances, this sharing occurred between pairs of samples from the same donor or pairs of samples that were adjacent at some point in the library preparation procedure. The shared cell barcode-VDJs were typically associated with an overall very large number of UMIs that were often concentrated in one of the samples (**Fig. S19**), indicating that they likely originate from antibody-secreting cells. This allowed us to identify one of the samples as the likely source of the contamination event. In a small fraction of cases, the shared cell-barcode VDJs were associated with a very small number of total UMIs and/or were more evenly distributed among the pair of samples (**Fig. S19**). These may represent true chance collisions of the cell barcode-VDJ pair, or may also be attributable to cross-contamination events but without a clear source sample. In this case, we removed the cell barcode-VDJ pair from further consideration. Conversely, when the shared cell barcode-VDJ was associated with a total of more than 100 UMIs, and when the fraction of UMIs in the secondary sample was smaller than 10^{-2}, we removed the pair from the secondary sample and labeled the primary sample as the "source" of the contamination event.

Given that shared cell barcode-VDJs were enriched for high-UMI-count VDJs when compared to cell barcode-VDJs that were private to one of the samples (**Fig. S20**), we wanted to investigate whether there might also be a large number of undetected cross-contamination events affecting low-UMI-count cell barcode-VDJs. Specifically, since most VDJs are associated with few recovered heavy chain UMIs per cell, we were concerned that the transfer of a small number of those molecules between samples might lead to the association of that VDJ with the incorrect sample. To quantify the expected number of cross-contamination events affecting low-UMI-count VDJs, we constructed a simple model of the contamination process. Assuming that each UMI has an equivalent probability μ of being transferred between a pair of samples that can be involved in a cross-contamination event, the probability of a cell barcode-VDJ with n total UMIs being transferred between two samples

(and passing filtration, which requires at least 2 UMIs to be transferred, see **Section C.2**), **Cell calling**, is

$$p_{\text{cont}}(n) \approx 1 - \exp[-\mu n] - \mu n \exp[-\mu(n-1)]. \qquad [1]$$

If there are $N(n)$ cells with n UMIs, then we expect that the total number of contamination events is equal to

$$\langle N_{\text{cont}}(n)\rangle = N(n)p_{\text{cont}}(n). \qquad [2]$$

Since cross-contamination can only occur between pairs of samples that are in physical proximity, to remain conservative, we limit the estimation of μ and $N_{\text{cont}}(n)$ to samples in which we identified probable contamination events (pairs of shared cell barcode-VDJs). We estimate μ to be equal to the ratio of the sum of secondary-sample-UMIs associated with shared cell barcode-VDJs and all of the UMIs present in the pair of samples, which yields a probability of transfer per UMI of $\approx 5.9 \times 10^{-5}$. Plugging into Equations 1 and 2, and plotting the result on **Fig. S20**, we find that our model estimates that cross-contamination events involving cells with a small number of UMIs should be extremely unlikely. Thus, we conclude that many of the low-UMI-count contamination events may indeed represent chance collisions of cell-barcodes between samples that contain identical VDJs, but that these VDJs represent such a small fraction of the sampled VDJs that they are unlikely to affect our results.

C.5. Transcriptome-informed analysis and annotation of ambient transcripts.

Abundance and distribution of ambient VDJ transcripts. After integrating our heavy chain VDJ transcript sequences and GEX data, we made use of the droplets associated with high-quality single non-B cells to assess the abundance of ambient VDJs in the environment. In almost all samples, a fraction of these droplets, ranging from 10^{-3} to about 0.15 contained VDJs derived from the ambient, with the majority of samples having ambient rates of a few fractions of a percent (see **Fig. S21**). The fraction of non-B cell droplets contaminated by an ambient VDJ transcript is correlated the number of high-abundance VDJ molecules in the sample (**Fig. S21**, right panel). Moreover, we find that samples thawed from storage in liquid nitrogen have no higher ambient rates than samples prepared fresh, but that there do appear to be other idiosyncratic factors that influence the rate at which the release of ambient transcripts occurs. These observations are consistent ambient antibody transcripts likely originating from antibody-secreting cells that have at least partially lysed prior to encapsulation in a droplet.

The scaling of the fraction of non-B cell droplets containing ambient VDJs with the number of high-expression VDJs also suggests that when such a lysis event occurs, it is typically limited to a small number of droplets, rather than being well-mixed in the reaction. This observation is also empirically apparent from the distribution of ambient VDJs among droplets in our data (**Fig. S22**): VDJs detected in a droplet-containing a non-B cell are typically distributed among a far smaller number of droplets than would be expected in a well-mixed model (**Fig. S22**, left panel), the majority of their UMIs are often found in a single droplet(**Fig. S22**, center panel), and more broadly, their UMIs are often highly unevenly distributed among droplets even in instances when the number of such droplets is large (**Fig. S22**, right panel). We emphasize that this spatial co-localization of ambient VDJ transcripts makes them in principle impossible to distinguish from cells in the absence of gene expression data. It also means that, in the absence of gene expression data, droplet counts are not good proxies for cell counts, and that presence of a VDJ in multiple droplets is not reliable evidence of clonal expansion.

These observations suggest that for many of these "ambient" VDJs, there might be an identifiable "source" cell, containing the majority of the VDJs and the transcriptome. Across our dataset, we find that 97% of all VDJ UMIs determined to be ambient via their association with a non-B cell transcriptome and 74% of all droplets containing an ambient VDJ can be traced back to an identifiable ASC or group of ASCs collectively carrying 78 unique VDJs. The remaining 258 ambient VDJs are typically supported by a smaller number of overall UMIs per VDJ and contain a wide diversity of VDJ sequences, without an identifiable ASC transcriptome in our dataset.

Finally, we were concerned that these types of lysis events may frequently affect other ASCs in our dataset but not end up encapsulated in non-B cell droplets, making them difficult to identify as ambient. However, if we only limit ourselves to droplets that also contain a high-quality single B cell transcriptome, we find that the UMIs associated with these VDJs are found in a single droplet in over 90% of cases, and in the cases in which they are identified in multiple B-cell associated droplets, they are notably more uniformly distributed across these droplets (see **Fig. S23**). We concluded that these signatures are rather suggestive of true clonal expansion among ASCs, and do not constitute clear evidence of lysis and spillage into a number of B-cell associated droplets.

The probability of GEX misidentification due to the presence of ambient VDJ transcripts. Ambient VDJs detected in non-B cells can be easily removed from further analysis, but their presence in droplets associated with true B cells may lead to the identification of the VDJ sequence with the incorrect transcriptome. This is especially a concern given that many VDJ transcripts do drop out in the library preparation process (see the left panel of **Fig. S25** and the discussion in **Section A.2**).

However, we can quantify the rate at which a VDJ transcript detected in a B cell droplet is actually ambient in origin. Specifically, the empirical rate, r_{total}, at which droplet with high-quality single B cell transcriptomes are found to also contain a VDJ transcript should be equal to the sum of the ambient contamination rates, r_{ambient}, and the rate at which the true rate is detected in that sample, r_{true}. Assuming that B cells are as likely as non-B cells to be associated with an ambient VDJ, the probability that the VDJ associated with the high-quality transcriptome is not ambient in origin is simply

$$P_{\text{ambientVDJ}} = \frac{r_{\text{ambient}}}{r_{\text{total}}}. \qquad [3]$$

We show the distribution of these probabilities for each unique GEX sample with at least 50 high quality non-B cells and for each cell type with at least 100 high quality B cells across all donors on **Fig. S25**. Though there is significant sample-to-sample variation in the rate at which VDJs associated high-quality B cell types drop out, we find that the ambient rates are low enough that for the overwhelming majority of samples, the probability that the identified VDJ is ambient is under 5% for all B cell subsets.

Finally, we constructed an orthogonal estimate of ambient VDJs by exploiting the fact that Naive B cells should not be associated with a class-switched IGHC segment. Indeed, as can be seen on **Fig. S24**, the vast majority of droplets determined to be associated with a single high quality Naive B cell transcriptome and a single heavy chain VDJ sequence are associated with a IGHM/D gene segment. In many samples, we can also detect a small fraction ($\lesssim 10^{-2}$) of such droplets that are associated with a class-switched IGHC gene segment. We hypothesized that these VDJs with class-switched IGHC gene segments derive from the ambient and sought to quantify whether their incidence is consistent with our previous estimate of ambient contamination rates (see Eq. 3).

Specifically, if we assume that all switched IGHC segments associated with Naive B cells droplets are ambient in origin, then for these droplets,

$$P(\text{switched}|\text{NaiveGEX}) = P(\text{switched}|\text{ambient}) \cdot P(\text{ambient}|\text{NaiveGEX}), \qquad [4]$$

from which it follows that

$$P(\text{ambient}|\text{NaiveGEX}) = \frac{P(\text{switched}|\text{NaiveGEX})}{P(\text{switched}|\text{ambient})}. \qquad [5]$$

Importantly, this approach allows for $P(\text{switched}|\text{ambient})$ to be smaller than 1, or, in other words, for IGHM/IGHD transcripts to also be ambient in origin, and is consistent with our observations of many antibody secreting cells and memory B cells being associated with unswitched isotypes (see **Fig. S4e** and **Fig. S5e**). We can estimate $P(\text{switched}|\text{ambient})$ empirically by once again using the VDJ sequences associated with droplets known to contain high quality single non-B cells, and assuming that it is equivalent to the switched fraction of those VDJs.

Annotation of ambient-derived VDJ transcripts. In samples for which we have GEX data, we annotated as 'ambient' VDJs associated with a high-quality non-B cell. In the small number of cases in which there was an identifiable ASC in the sample with the same VDJ, we annotated that cell as a 'source' of ambient material in cases in which the droplet in which the cell was encapsulated:

1. contained the largest number of UMIs mapping to that VDJ of all droplets that contained that VDJ,
2. contained more than 500 UMIs supporting that VDJ, and
3. accounted for more than 25% of the overall number of UMIs associated with that VDJ in that sample.

Quantification of per-sample VDJ multiplicity per droplet. The high diversity of heavy chain transcripts in our samples offers an avenue for the quantification of the rate at which two or more recovered VDJ sequences coincide in the same droplet (i.e. the rate of VDJ "multiplicity"). After the removal of cross-contaminants and ambient transcripts as described above, we quantified the fraction of cell barcodes with any VDJ sequences associated with them that had a single VDJ sequence associated with them. In all samples loaded at a target of 20,000 cells per lane ("VDJ + GEX"), as well as in the vast majority of samples loaded at a target of 80,000 cells per lane("VDJ-only"), a majority of droplets contain only a single VDJ sequence (see **Fig. S26**).

In all analyses sensitive to cell counting, as well as in all analyses in which we associate transcriptomes with VDJ sequences, we only use droplets associated with a single VDJ sequence, and discard droplets with two or more VDJs. Note that such analyses also exclude all data from VDJ-only samples, because we determined that the presence of a VDJ sequence in a droplet represents a poor proxy for a cell in a droplet in the absence of transcript information (see **Section C.5**, **Abundance and distribution of ambient VDJ transcripts**, second paragraph). This difficulty does not stem from the loading rates themselves, but rather from the fact that cell lysis and encapsulation happen on similar timescales in this experimental implementation of emulsion-based single cell sequencing, and highly expressed immunoglobulin transcripts can be partitioned onto multiple droplets during this procedure. Thus, we only use VDJs from these samples in analyses insensitive to cell counts, such as those in which we simply quantify the number or fraction of shared unique VDJ sequences, treating these samples as pseudo-bulk samples.

We still find the use of encapsulation to be advantageous in these samples (as compared to bulk sequencing) since it allows for the correction of any errors that arise during reverse transcription. Such errors may lead to particularly problematic insights in the context of samples containing a mixture of ASCs and memory B cells, where the extremely high expression of immunoglobulin transcripts in ASCs may lead to frequent reverse transcription errors being misidentified as closely related memory B cells.

D. Analysis of heavy chain sharing between donors. VDJ nucleotide sequence sharing between donors is remarkably rare (6 in over 600 000 unique VDJ sequences, see **Fig. S9a**), which justifies our use of the VDJ nucleotide sequence as a unique clonal identifier within a donor. When VDJs that are shared between donors do arise, they are always seen in cells in which we do not see any hypermutations in the templated V sequence (see **Fig. S9b**). This suggests that are associated with commonly generated CDR3s arising in independent recombination events, as opposed to convergently evolved antibodies arising in response to a common antigen. Notably, the absence of shared hypermutated VDJ nucleotide sequences suggests that even

convergent recombination is far too rare an occurrence compared to the diversifying force of hypermutation to lead to the repeated generation of the identical heavy chain nucleotide sequence within the same individual. Note that this definition departs from the more commonly used definition of a "clonotype" (for heavy chains with an identical V-gene, J-gene and CDR3 amino acid sequence (13), and that it represents a far more stringent clonal identifier (**Fig. S9c**).

To verify that our shared VDJ nucleotides sequences indeed represent convergent recombinants in naive cells, we used OLGA to calculate CDR3 generation probabilities for all CDR3s associated with unhypermutated V-gene segments(14). Since the calculation of generation probabilities of hypermutated CDR3s also requires a model of hypermutation and selection in the germinal center reaction, which is not currently known in full, we further removed from consideration (for the purposes of this Section) all lineages in which we identified multiple unique CDR3 nucleotide sequences associated with unhypermutated VDJs. These species certainly contain hypermutated CDR3, and would lead to the erroneous inference of the generation probabilities for that CDR3, possibly skewing the entire distribution towards lower-probability CDR3's. As we show on **Fig. S9d**, CDR3s found in a single donor have a wide variation of generation probabilities, as inferred by OLGA. In contrast, CDR3s associated with multiple donors have exceptionally high generation probabilities, as do CDR3s associated with the 6 instances of shared VDJ nucleotide sequences between donors.

We calculated how our sharing statistics compare to those predicted by this empirical distribution of generation probabilities. To do this, we broadly followed the approach from Ref. (15), but with a slight departure in our enumeration of the number of independently generated CDR3s in our dataset. In Ref. (15), the authors, dealing with bulk TCR sequencing data, take the number of unique CDR3 amino acid sequences as a proxy for the number of unique recombination events. Here, we take the number of lineages associated with unique CDR3 nucleotide sequences and unhypermutated V genes to be the number relevant number independent rearrangements. We compare our sharing statistics to two versions of the sharing model, as in Ref. (15). First we calculated the expected amount of sharing assuming that the probability that a productive CDR3 sequence passes negative selection at the pre-B cell stage to be order 1 (see dashed line, **Fig. S9e**). We find that this results in comparable, but slightly lower levels of sharing than seen in our data. Therefore, we concluded that it is likely that a much smaller percentage of productive CDR3's support high-enough quality antibodies to pass negative selection. This observation is consistent with a similar effect seen for TCRs, where only 3.7% of all productive CDR3s pass thymic selection (15). Thus, we used our data to estimate an analogous "selection factor" for B cells, by fitting the total number of unique amino acid CDR3 sequences, conditioned on the total number of sampled independent recombination events (lineages) in each donor. We find that this factor is variable among donors, likely reflecting the paucity of data for this type of estimation, but using the mean inferred selection factor of 1.8%, we find that the empirical number of shared CDR3s lies between the two extremes of the two models we considered.

E. VDJ and lineage sharing analysis.

E.1. VDJ sharing between tissues and subanatomical regions. To construct a fair estimate of the level of VDJ sharing between all pairs of tissues and subanatomical regions, we worked to account for sample-to-sample variability in the depth of B cell sampling in our experiment by downsampling all independently generated emulsions (i.e. all independent 10X lanes) or "samples" (in the remainder of this section) to an equivalent number of 3000 unique VDJs prior to performing any comparisons. We chose this number so as to maintain the largest possible number of comparisons between pairs of tissues, and thus dropped the few extremely low-yield emulsions that contained fewer than 3000 unique VDJs from consideration in this analysis. In each sample, we obtained 100 independent samples of groups of 3000 unique VDJs, weighing each of the originally sampled VDJs equivalently (i.e. independently of the number of UMIs or droplets that the VDJ is associated with).

For each pair (t_1, t_2) of tissues and subanatomical regions (i.e. different individual lymph nodes), we report two quantities, the unscaled probability $p(t_2|t_1)$ and the scaled probability $p_{\text{normalized}}(t_2|t_1)$, which we calculate as we describe below.

First, to calculate the unscaled probability that a VDJ sequence found in tissue t_1 is also present in tissue t_2 $p(t_2|t_1)$, we simply calculate the average fraction of the VDJs found in tissue t_1 that are also found in tissue t_2, averaging over all possible pairs of t_2-t_1 samples derived from independent emulsions, and all replicates of each sample

$$p(t_2|t_1) = \left\langle \frac{1}{100} \sum_{\text{replicate}=0}^{100} \frac{\sum_{VDJ \in t_1} I_{\text{VDJ}}(t_2)}{\sum_{VDJ \in t_1} I_{\text{VDJ}}(t_1)} \right\rangle_{\text{available } (t_1, t_2) \text{ pairs of samples}}, \tag{6}$$

where the indicator variable $I_{\text{VDJ}}(t)$ denotes membership of the VDJ sequence in the sample of tissue t.

In many cases, VDJs that are truly shared between pairs of tissues may be missing simply because we are only sampling a small fraction of all the VDJs in the tissue in each sample. To account for this loss due to sampling, we scaled the raw probability $p(t_2|t_1)$ with the probability that a VDJ found in a sample of tissue t_1 is also found in an independent sample (i.e. an independent emulsion) of the same tissue (which we denote with t_1'). Crucially, we compute the average of the ratio of the quantities, not the ratio of the average:

$$p_{\text{normalized}}(t_2|t_1) = \left\langle \frac{1}{100} \sum_{\text{replicate}=0}^{100} \frac{\frac{\sum_{VDJ \in t_1} I(t_2)}{\sum_{VDJ \in t_1} I(t_1)} + 0.1}{\frac{\sum_{VDJ \in t_1} I(t_1')}{\sum_{VDJ \in t_1} I(t_1)} + 0.1} \right\rangle_{\text{available } t_1', t_1, t_2 \text{ combinations of samples}}. \tag{7}$$

We also refer to this quantity as the“normalized" probability. We note that in the event that the distributions of the fraction of clonal cells in the tissue that carry the same VDJ are equivalent in the two tissues, and that the sampling biases during the data generation process are equivalent for the two tissues, this procedure truly scales out the sampling probability. However, even if that condition is not met, this procedure effectively conditions on the VDJ being present in a sufficiently high fraction of all cells in tissue t_1 to be repeatedly sampled in independent samples of 3000 unique VDJs.

We report these quantities alongside their standard errors, computed from the variability among the different independent pairs of emulsions from tissues t_1 and t_2.

Finally, we also calculated the raw and scaled probabilities of discovering a VDJ in a sample of tissue t conditioned on it being present in a sample of two or more tissues $\{t_i\}$ in an analogous way:

$$p(t|\{t_i\}) = \left\langle \frac{1}{100} \sum_{\text{replicate}=0}^{100} \frac{\sum_{VDJ \in \{t_i\}} I_{\text{VDJ}}(t)}{\sum_{VDJ \in \{t_i\}} I_{\text{VDJ}}(\{t_i\})} \right\rangle_{\text{available } (t,\{t_i\}) \text{ combinations of samples}}, \quad [8]$$

and

$$p_{\text{normalized}}(t_2|\{t_i\}) = \left\langle \frac{1}{100} \sum_{\text{replicate}=0}^{100} \frac{\frac{\sum_{VDJ \in \{t_i\}} I(t)}{\sum_{VDJ \in \{t_i\}} I(\{t_i\})} + 0.1}{\frac{\sum_{VDJ \in \{t_i\}} I(t_i')}{\sum_{VDJ \in \{t_i\}} I(\{t_i\})} + 0.1} \right\rangle_{\text{available } \{t_i\},t_i',t \text{ combinations of samples}}, \quad [9]$$

where t_i' refers to an independent replicate of one of the tissues t_i on which the presence of the VDJ was conditioned.

We find that the normalized probability of sharing obeys a simple frequency dependence consistent with there being a constant per-cell probability of exit from tissue t_i into a second tissue t_j. If we denote the inverse of this probability with $m_{i \to j}$ then the probability that a lineage with n_i cells has in tissue t_i has any members in tissue t_j is

$$p_{\text{present,j}}(n_i) = 1 - e^{-m_{i \to j} n_i}. \quad [10]$$

Since we cannot measure the absolute size of a lineage n_i, but only its fractional abundance in the tissue, it is more convenient to rewrite this probability in terms of that fractional abundance. We denote this quantity with x_i, so that $x_i = n_i/N_i$, where N_i is the total number of B cells in tissue t_i. We also define the characteristic frequency scale for the exit from tissue i into tissue j, $x_{c,i \to j}$, as

$$x_{c,i \to j} = \frac{N_i}{m_{i \to j}}. \quad [11]$$

Rewriting in terms of the lineage fractional abundance x_i, we obtain

$$p_{\text{present,j}}(x_i) = 1 - e^{-x_i/x_{c,i \to j}}. \quad [12]$$

For each pair of tissues for each donor, we infer $x_{c,i \to j}$ directly from the data using a simple least squares fit of Eq. 12 to Eq. 9. We implemented this by using the `curve_fit` method from the `scipy.optimize` module.

The scaled probabilities of appearance of a lineage in a second tissue, broken out by the second tissue, are shown in **Fig. S27**.

E.2. Enrichment of cell types among shared VDJs. We were interested in understanding the extent to which cells in different functional states were enriched among the cells carrying shared VDJs. A VDJ was considered associated with a cell type if it was determined to be associated with a droplet containing that cell type in any of the tissues in which it was sampled.

To evaluate the enrichment in the amount of shared cells associated with a specific VDJ sequence, we constructed a simple null model that assumes that shared VDJs are not likely to be associated with any cell type in particular. This simultaneously represents a conservative model of ambient-RNA contamination of droplets of a certain cell type. In our null model, we would the expectation of the fraction of VDJs found associated with a certain cell type c and found in only a single tissue t $\langle f_{\text{single,c}}(t) \rangle$ to be equal to the total fraction of cells of that cell type in the tissue, $f_c(t)$:

$$\langle f_{\text{single,c}}(t) \rangle = f_c(t). \quad [13]$$

The null expectation of the fraction of VDJs found associated with a certain cell type in and found in a pair of tissues t_1 and t_2 is equal to

$$\langle f_{\text{pair,c}}(t_1, t_2) \rangle = 1 - (1 - f_c(t_1))(1 - f_c(t_2)). \quad [14]$$

To construct the expected fraction seen in any single tissue or any pair of tissues of the same donor, we computed a weighted mean of the individual single tissue and tissue pair null expectations, where the weight was equivalent to the total number of observed cells in that tissue or tissue pair. Finally, for each quantity, we report the simple average across all donors.

E.3. Gene expression associated with shared VDJs. We explored gene expression signatures in cells with shared VDJ sequences across tissues or subanatomical regions. We started by identifying genes which were differentially expressed between shared and non-shared cells, and subsequently evaluated the discriminative power of these genes. To perform differential expression analysis, we labeled each cell's gene expression profile as "shared" between tissue pairs if its associated VDJ sequence was detected in another tissue within the same donor. We then used the Wilcoxon rank-sum test to identify genes which were differentially expressed between shared or not shared cells in each pair of tissues. In all pairs of tissues, the genes identified were indicative of an proliferative ASC phenotype, corroborating our findings in **Section E.2**.

We were interested if there was a signature associated with the multi-tissue presence of memory B cell clones. To evaluate this, we performed the same differential expression analysis on only the memory B cells. For illustration, the top 12 differentially expressed genes for the Lymph Nodes and the Spleen are shown **Fig. S11a**). Upon considering other pairs of tissues, we noticed that many of them had similar sets of differentially expressed genes . Thus, we used the 45 most differentially expressed genes between shared memory B cells in the Lymph Nodes and Spleen as the features to train a logistic regression classifier for sharing across all tissue pairs. Before training, we downsampled the data to remove the class imbalance in the sharing variable, because a large majority of cells were not shared. We found that this classifier could be used to enrich for shared memory B cells across any pair of tissues, suggesting that there is a common gene expression profile associated with memory B cell clones present in multiple tissues (**Fig. S11c**), though it is difficult to report a measure of the false-negative and false positive rates associated with it, because many truly shared VDJs will not have a 'shared' label in the context of limited sampling. We report the inferred scale parameters ('feature importances') for each of the features included in the classifier in **Fig. S11b**.

E.4. Lineage sharing between tissues. We quantified the level of lineage sharing between tissues using an entirely analogous approach to the one we used to quantify VDJ sharing described in **Section E.1**, with two minor differences:

1. all samples were downsampled to 5000 unique VDJs prior to estimating all quantities, and samples in which we recovered fewer than 5000 unique VDJs were dropped from the analysis. The reported quantities represent averages of all possible pairs of samples informing the comparison between two tissues, and were computed based on a single random subsample, and

2. we computed normalized probabilities of sharing of lineages conditioned on their binned frequency in one of the tissues.

We emphasize that, with the exception of TBd1, in whose peripheral blood and bone marrow we find evidence of a substantial excess of high-frequency lineages, lineage size distributions collapse after subsampling, justifying the scaling procedure used above to account for subsampling.

F. B cell differentiation during affinity maturation. In this section, we consider in more detail the dynamical process of differentiation within a germinal center. We start by considering a simple model in which all cell fate decisions are constant in time, and show that this model results in a uniform distribution of hypermutation levels of all cell types within a lineage. We explain why differences in exponents necessarily imply differences in exit rates between different lineages and then then turn to quantifying the empirical per-cycle exit rates of all cells.

F.1. A constant rate model of B cell differentiation. In the simplest model of the GC reaction that is consistent with the observation of uniform distributions of hypermutation levels, the GC reaction proceeds as a memory-less process in which all cell fate decisions are constant in time. To elaborate, in each round of selection in the light zone of the germinal center, GC B cells may differentiate and exit the GC as Memory B cells or ASCs, reenter the dark zone for continued hypermutation, or fail to receive signals for continued survival and proliferation and undergo apoptosis. Crucially, the probability of each of these events does not change as the GC reaction proceeds. We illustrate this process schematically on **Fig. S28**.

Shortly after establishment, let the germinal center contain naive cells entering the dark zone of the germinal center, where they each undergo several divisions. After each round of proliferation in the dark zone, the descendants of these naive cells experience selection in the light zone, upon which they have the opportunity to differentiate, die, or re-enter the dark zone. The full specification of this model requires us to also specify a model of proliferative bursts in the dark zone of the GC, i.e. the total distribution of the number of descendant cells that exit the dark zone for each entrant to the dark zone, and the distribution of the number of new hypermutations they acquire, as well as an explicit model of competition. In principle, these quantities may vary between cells in a way that correlates with their differentiation potential and also, possibly, previous proliferative bursts. A broad consideration of this class of models is beyond the scope of this work.

However, we can still make conceptual progress by making several simplifying approximations. Assuming that a B cell accumulates on average V hypermutations in the templated portion of the IGHV gene in each germinal center cycle and that its descendants have a constant net per-cycle rate of exiting the germinal center cycle either by dying (at per-cycle rate δ) or by differentiating (at per-cycle rate θ_k into each of the cell types k), the probability of germinal center exit in each cycle is simply

$$\varepsilon = \delta + \sum_k \theta_k. \qquad [15]$$

Conversely, the probability of the descendants of the cell remaining in the germinal center reaction and acquiring another V hypermutations is $1 - \varepsilon$. Thus, the probability that an exit from the germinal center reaction happens after exactly n cycles is,

$$P(\text{exit}, n\,\text{cycles}) = (1-\varepsilon)^{n-1} \cdot \varepsilon. \qquad [16]$$

When such an exit occurs, the total fraction of events that result in differentiation into cell type i is

$$P(i|\text{exit}) = \frac{\theta_i}{\varepsilon}. \quad [17]$$

Combining these two expressions and noting that when the number of cycles n is large we can approximate the total number of hypermutations m as $n \cdot V$, we obtain that the total probability of differentiation into celltype i after acquiring m hypermutations is

$$P(i, m\,\text{hypermutations}) = \theta_i (1-\varepsilon)^{m/V-1} = \theta_i e^{\log(1-\varepsilon)\left(\frac{m}{V}-1\right)}. \quad [18]$$

This model generates two important predictions. The first is that, among the events that result in differentiation, the relative probability that an emerging differentiated cell has exited as a specific cell type i is simply

$$P(i|\text{differentiation}) = \frac{\theta_i}{\sum_k \theta_k}. \quad [19]$$

This quantity does not depend on the number of elapsed cycles or the total number of hypermutations that have accumulated. Thus, cells of different hypermutation levels have equal relative probabilities of emerging at any point in the germinal center reaction. This prediction is consistent with our observation of uniform distribution of hypermutation levels among related cells of different types.

The second prediction of the model is that the distribution of hypermutation levels among cells of the same type should be exponential with the same rate and should only depend on the total per-cycle exit rate ε and the number of hypermutations accumulated in each cycle V (see Eq. 18). Though we have seen that the distribution of hypermutation levels among cells of the same type does have an exponential tail, the rate of these exponents are different.

F.2. Empirical quantification of repertoire-wide exit rates for different cell types. Though differentiation cells of different types are uniformly distributed within lineages, we see notable repertoire-wide differences in the amounts of hypermutations visible in cells of different types. This can only arise if the repertoire-wide per-cycle exit rates, ε, or the repertoire-wide per-cycle hypermutation rates V are different for cells of different types, since those are the quantities that control the exponents of the hypermutation distribution. However, since we observe no differences in the distributions of hypermutations of related cells, this must imply that different lineages must have different death and/or differentiation rates. Differences in these parameters between lineages could either arise as a result of a form of statistical contingency in the binding landscape of the antibody, an intrinsic property of the initial cell state, or due to a contingency in the form of differentiation signals available to that cell in the germinal center (e.g. the availability of certain cytokines, or the specificity of the available T cells in the germinal center). These different processes are not identifiable from our data.

However, this conclusion raises the empirical question of whether or not there is a repertoire-wide temporal switch in the rates at which cells of different subtypes emerge. To quantify this, we define the notion of effective exit rates, $\varepsilon_i(t)$, which represents the repertoire-wide rate at which cells of type i emerge from the germinal center reaction after t cycles. With this definition, the probability of exiting with m or more hypermutations is

$$P_i(\geq m\,\text{hypermutations}) = \prod_{t=0}^{\lfloor m/V \rfloor - 1} [1 - \varepsilon_i(t)] = \exp\left(\sum_{t=0}^{\lfloor m/V \rfloor - 1} \log(1-\varepsilon_i(t)) \right). \quad [20]$$

These can therefore be explicitly obtained from the derivatives of the CDF of the hypermutation distribution. Specifically, for large enough m that we can approximate the sum with an integral and that $\lfloor \frac{m}{V} \rfloor - 1 \approx \frac{m}{V}$,

$$-\frac{d}{dm} \log P_i(\geq m\,\text{hypermutations}) \approx -\frac{1}{V} \log(1 - \varepsilon_i(m)). \quad [21]$$

Note that for small exit rates, $\varepsilon_i(m) \ll 1$, $-\log(1-\varepsilon_i(m)) \approx \varepsilon_i(m)$, motivating the identification of Eq. 21 with the per-hypermutation exit rate.

In the special case in which $\varepsilon_i(m)$ is constant and equal to ε_i, then at sufficiently large m,

$$P_i(\geq m\,\text{hypermutations}) \propto (1-\varepsilon_i)^{m/V}, \quad [22]$$

and the negative logarithm of the complementary cumulative density function (CDF)) is expected to increase linearly with the number of hypermutations m

$$-\log\left(P_i(\geq m\,\text{hypermutations})\right) = m \frac{\log\left(\frac{1}{1-\varepsilon_i}\right)}{V} + C \equiv k_i m + C_i \quad [23]$$

with slope k_i that is related to the exit rate ε_i according to

$$\varepsilon_i = 1 - \exp(-k_i V). \quad [24]$$

We compare the predictions of the expectation in Eq. 24 with the distributions of hypermutation levels in our data in **Fig. S29**. The inferred slopes k_i and the corresponding per-cycle exit rates ε_i obtained from k_i assuming that cells undergo on average 1

division per cycle ($V \approx 0.3$, (16, 17)) are shown in **Table S3**. Notably, all of the inferred slopes, and therefore also the exit rates, ε_i, are very similar, but not identical.

In **Fig. 4d** in the Main Text we plot the ratio of the empirical exit rates from Eq. 21 with the long-term exit rates per cell type tabulated in **Table S3**. We find that the two quantities are the same for Memory B cells and ASCs that have accumulated at least 10 hypermutations. However, we also find a modest reduction in the number of weakly hypermutated memory B cells and ASCs compared to the expectations of this model. The rate at which early ASCs exit from the germinal center is similar for ASCs of different types and lower than that for memory B cells by up to a factor of 2, but becomes indistinguishable for the differentiated B cell types after they have accumulated about 10 V gene hypermutations (corresponding to about 3% divergence from germline). We note that in lineages that are sufficiently large for us to be able to make comparisons between related cells, the relative fraction of cells with fewer than 10 V gene hypermutations is very small. Specifically, among lineages for which more than 3 unique VDJs have been sampled in our dataset fewer than 20% of all VDJ sequences have fewer than 10 hypermutations, and so the majority of the cells in these lineages have exited the germinal center at a time when our data indicate differentiated cells exit it with a constant probability at every GC cycle (**Fig. S30**).

We propose two potential hypotheses for the absence of weakly hypermutated differentiated B cells. First, it is possible that cells destined to become differentiated B cells undergo a larger proliferative burst in the dark zone of the germinal center, acquiring potentially a larger number of hypermutations in each GC cycle (corresponding to a larger V parameter in our model, since proliferation and hypermutation are coupled). This alone would make the exit of a differentiated B cell at a small number of hypermutations less probable, since return to the light zone is thought to be necessary for differentiation signals. Moreover, the proliferative burst size and therefore the number of hypermutations acquired in each cycle may vary between the different exit states, and may be lower for memory B cells than for ASCs. However, as long as the distribution of these burst sizes for cells of the same type is not too broad, we expect this model to yield a distribution indistinguishable from the one considered above at large m.

Second, it remains possible that at very early stages of the GC reaction, there remains explicit time-dependence in the rate at which GC cells differentiate into memory B cells and ASCs, as proposed before by other authors (see Ref. (18)). If this is the case, our data suggests that this time-dependence is limited to very early stages of the GC reaction, and that 80% of all differentiated cells exit the GC at times when this effect is negligible.

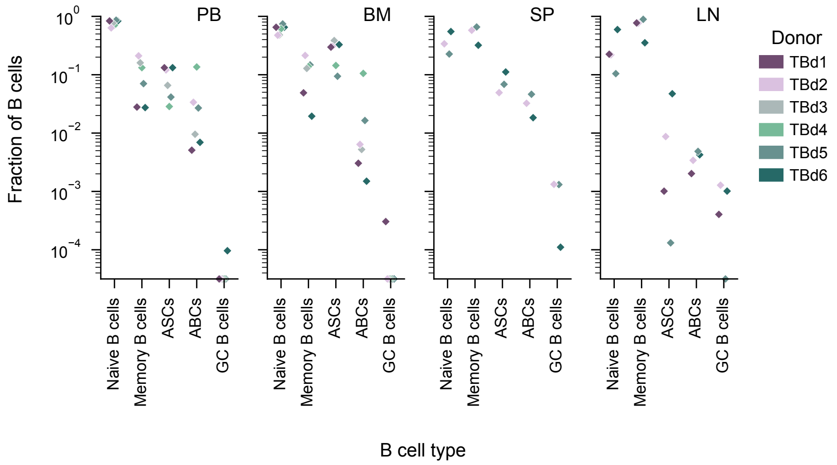

Fig. S1. B cell type distribution across donors and tissues. Displayed are cell states represented by more than 5 cells across all donors. Pre and pro B cells are discussed in **Section B.5**. In cases in which a cell type in a sampled tissue was not detected among the sampled B cells, the symbol for that donor is shown on the x-axis. ASC - antibody secreting cell, ABC - atypical B cell, GC B cell - germinal center B cell.

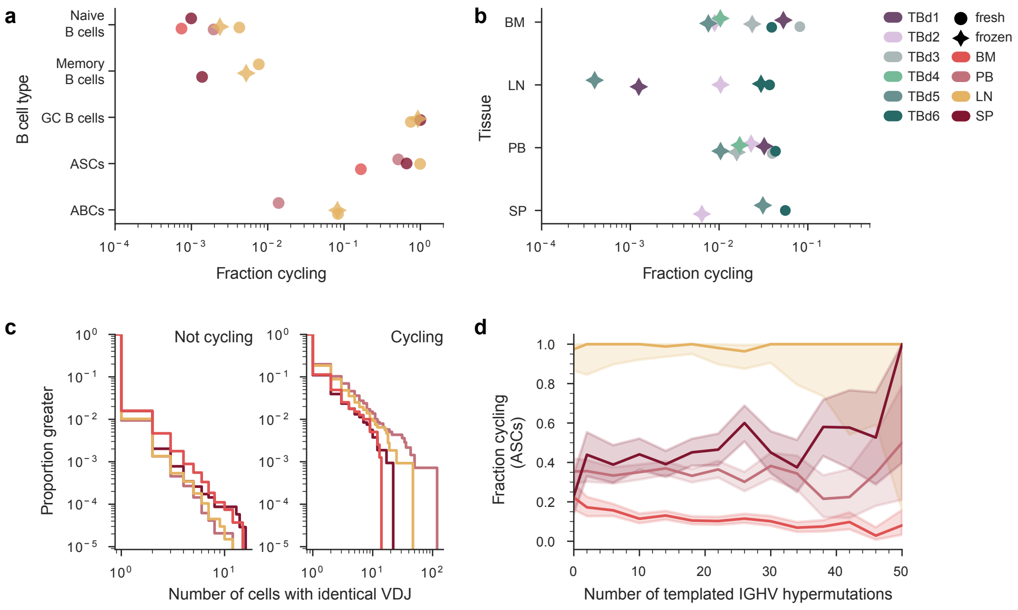

Fig. S2. Cycling phenotype across tissues and cell types. (a,b) Fraction of cells of each cell type and tissue (a), and tissue and donor (b) that are cycling. Markers denote whether a particular sample of a tissue was prepared fresh or previously cryopreserved. (c) The distribution of numbers of cells with identical VDJ sequences plotted by whether any or none of the sampled cells is found to be cycling. (d) Fraction of ASCs that are cycling shown for different tissues across hypermutation levels. Shaded areas denote the 95% confidence intervals are binomial proportion intervals calculated using the Clopper-Pearson method. Colors as in (b)

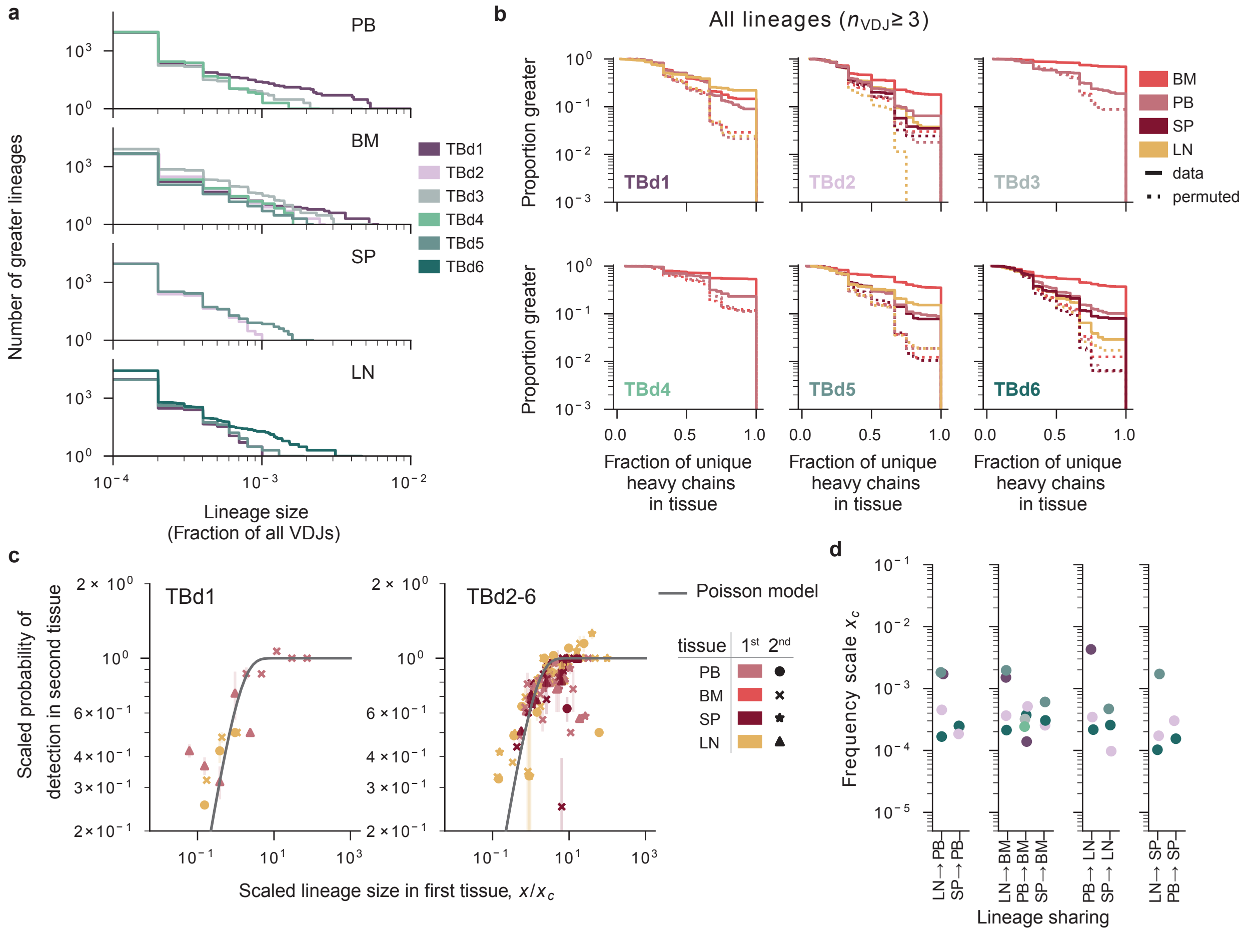

Fig. S3. Distributions of cell types among cells belonging to the same lineage. (a) Lineage size distributions for the data used in **Fig. 2c** in the Main Text (b) The distribution of the fraction of unique VDJs in the lineage found in a certain tissue. Each panel is a different donor. Dotted lines denote the distributions obtained after a random permutation of the tissue labels within the same donor among the sampled cells. Each tissue has been down-sampled to the same overall number of cells. (c) The scaled probability that a lineage is discovered in a tissue, with recently vaccinated donor TBd1 separated. Data plotted as in **Fig. 2c** in the Main Text. (d) Inferred frequency scale x_c from Main Text **Fig. 2c** for each between pairs of organs where point are estimates for each donor (colors as in (a)).

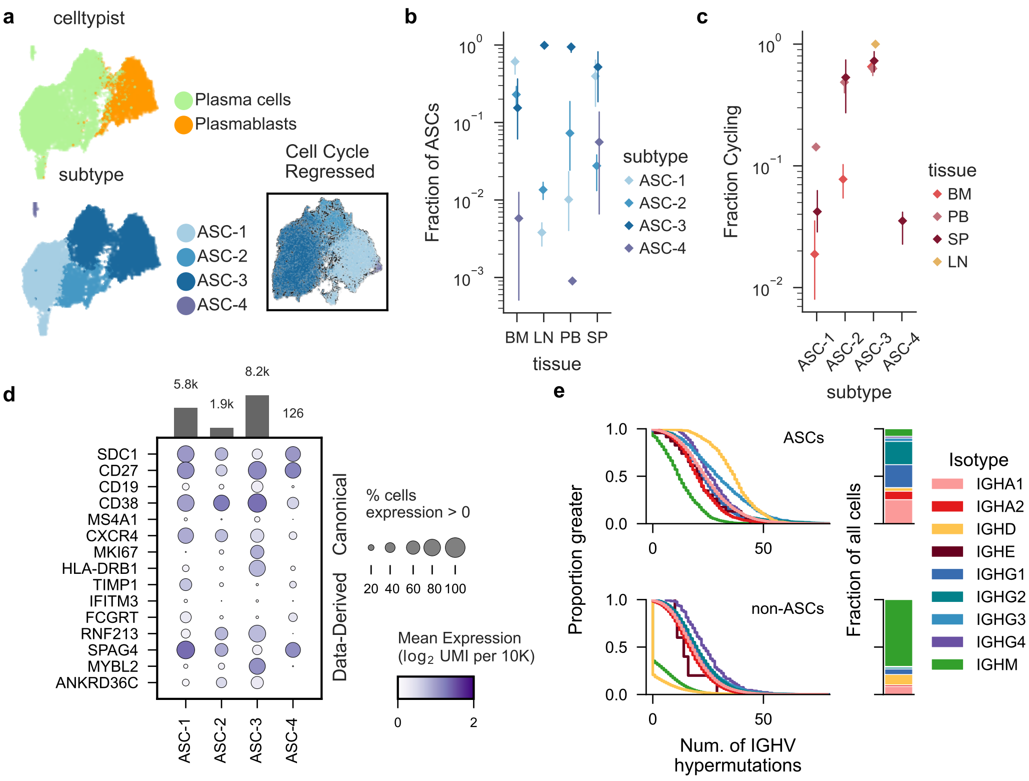

Fig. S4. Characterization of ASC transcriptomes and VDJs. (a) Low-dimensional (UMAP) representation of transcriptional heterogeneity in ASCs. Celltypist labels (top), and transcriptome-derived labels (bottom). Inset shows UMAP representation when cell-cycle associated genes are removed. (b) The relative fractional abundance of ASC subtypes in different tissues. Points represent means across all donors and error bars represent the range observed among the different donors. (c) Fraction of each ASC subtype that is cycling in each tissue. Data contributes to the plot if there are more than 30 ASCs in the sample and there are more than 10 ASCs of the relevant grouping (i.e. more than 10 ASC-1 detected in a 10X lane). (d) Genes distinguishing between the subtypes of ASCs, where the top half of the plot shows canonical genes often used for flow cytometry and the bottom shows the data-derived, transcriptionally detected genes which distinguish the subtypes. (e) The distributions of hypermutation levels for ASCs and non-ASC B cells of different isotypes (left) and overall isotype usage for ASC and non-ASC B cells.

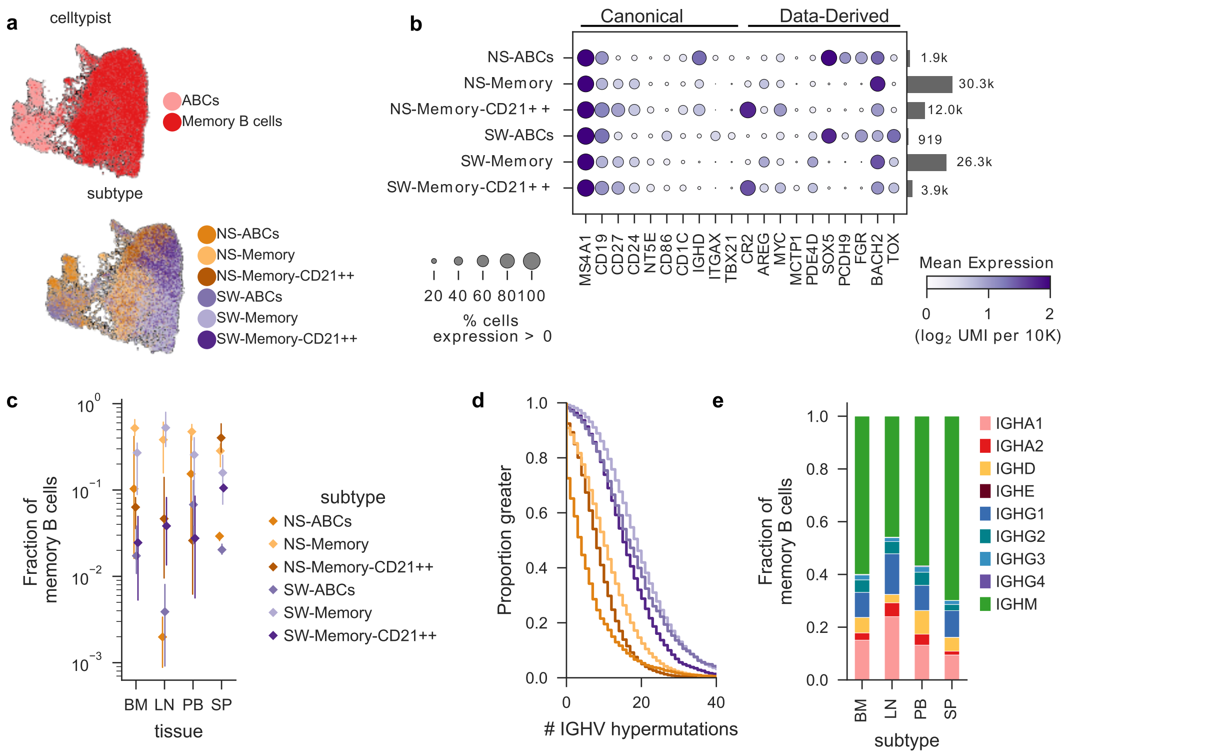

Fig. S5. Characterization of in memory B cell transcriptomes and VDJs. (a) Low-dimensional (UMAP) representation of transcriptional heterogeneity in memory B cells. Celltypist labels (top) and transcriptome-derived subtype labels (bottom). For these UMAPs, cell types that account for more than 1% of the population were sampled uniformly before calculating the nearest-neighbor graph. (b) Genes distinguishing between the subtypes of memory B cells, where the right half of the plot shows canonical genes often used for flow cytometry and the left shows the data-derived, transcriptionally detected genes which distinguish the subtypes. (c) The relative fractional abundance of memory B subtypes in tissues averaged across all donors. Points represent means across all donors and error bars represent the range of the data (d) The distribution of hypermutation levels for memory B cell subtypes. (e) Barplot of constant region usage in tissues for all memory B cells.

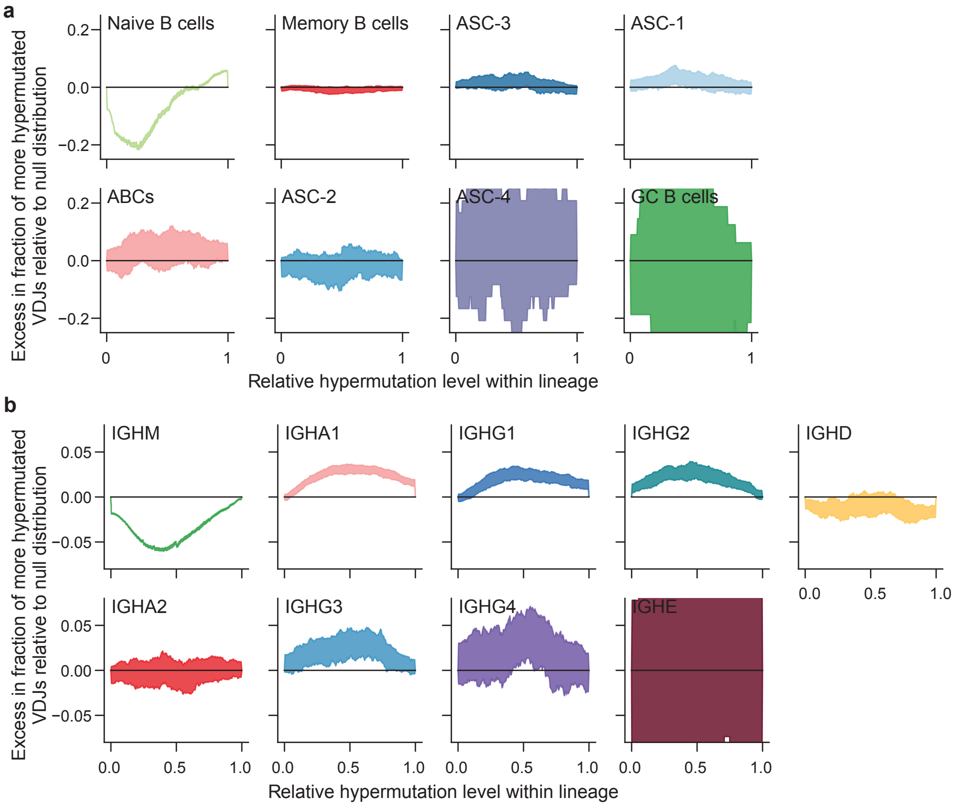

Fig. S6. Distributions of cell types and isotypes among cells belonging to the same lineage. (a) The within-lineage distribution of cells of a particular cell type. The panels corresponding to Naive B cells, Memory B cells, and ASC1-3 and duplicated from **Fig. 4b** in the Main Text, and are shown here separately for visual clarity. (b) The distribution of hypermutation levels for all cells in lineages with any cells using a particular constant region. As in Main Text **Fig. 4b**, the scaled hypermutation level represents the ratio of the excess in the number of hypermutations relative to the least hypermutated sequence in the lineage, and the excess number of hypermutations of the most hypermutated sequence in the lineage. Null distributions were obtained by permuting IGHC gene labels within lineages. The confidence intervals represent the bounds on the cumulative density function obtained in a 100 independent permutations.

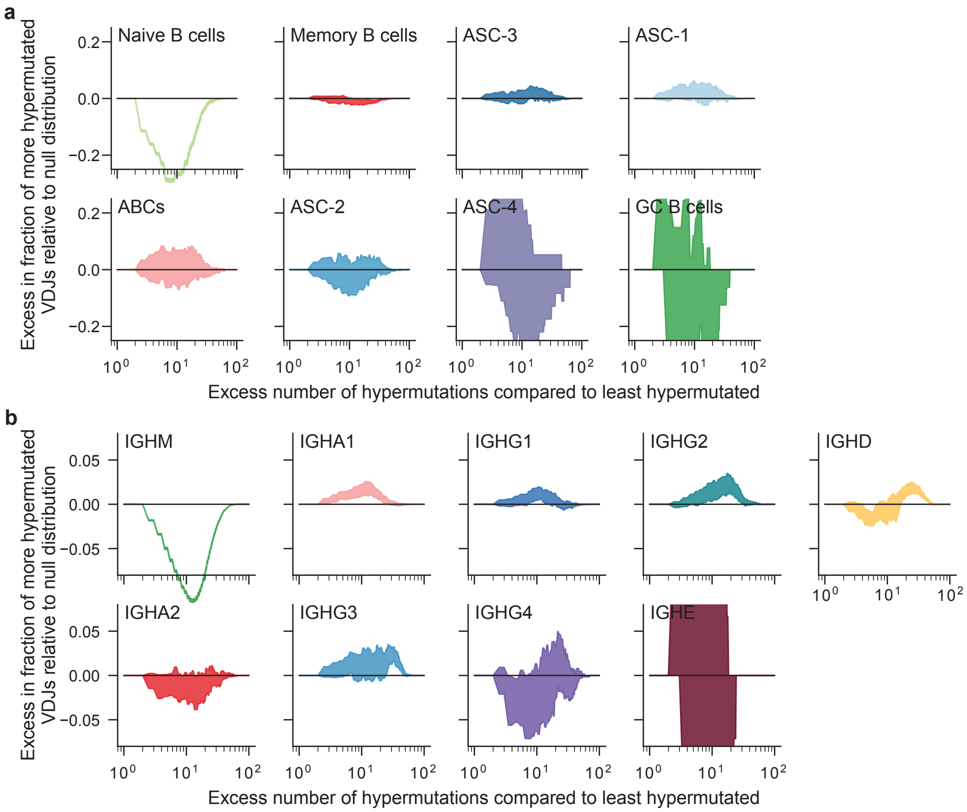

Fig. S7. Distributions of cell types and isotypes among cells belonging to the same lineage. (a) The within-lineage distribution of cells of a particular cell type. (b) The distribution of hypermutation levels for all cells in lineages with any cells using a particular constant region. As in, **Fig. S6**, but with the relevant statistic being the raw number of hypermutations relative to the least hypermutated sequence in the lineage. Null distributions were obtained by permuting IGHC gene labels within lineages. The confidence intervals represent the bounds on the cumulative density function obtained in a 100 independent permutations.

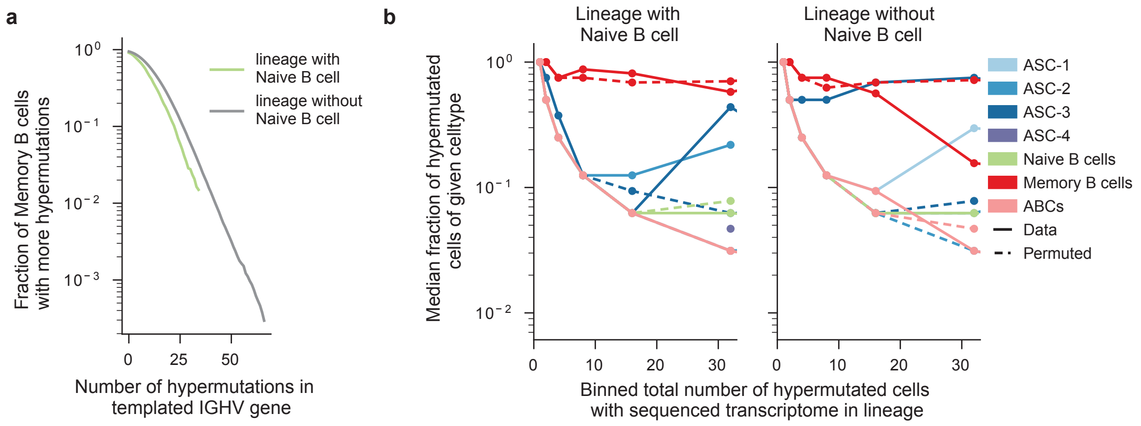

Fig. S8. Correlations in cell types of cells belonging to the same lineage. (a) Distribution of the of IGHV hypermutations in all memory B cells (grey) and memory B cells found in the same lineage as an unhypermutated Naive cell (green). (b) Average fraction of cells of given type in lineages in which an unhypermutated Naive cell was sampled (left), and in lineages in which none of the sampled cells were an unhypermutated Naive cell (right). Full lines represent data, and dashed lines represent distributions obtained after permuting cell state labels among all cells with sequenced transcriptomes.

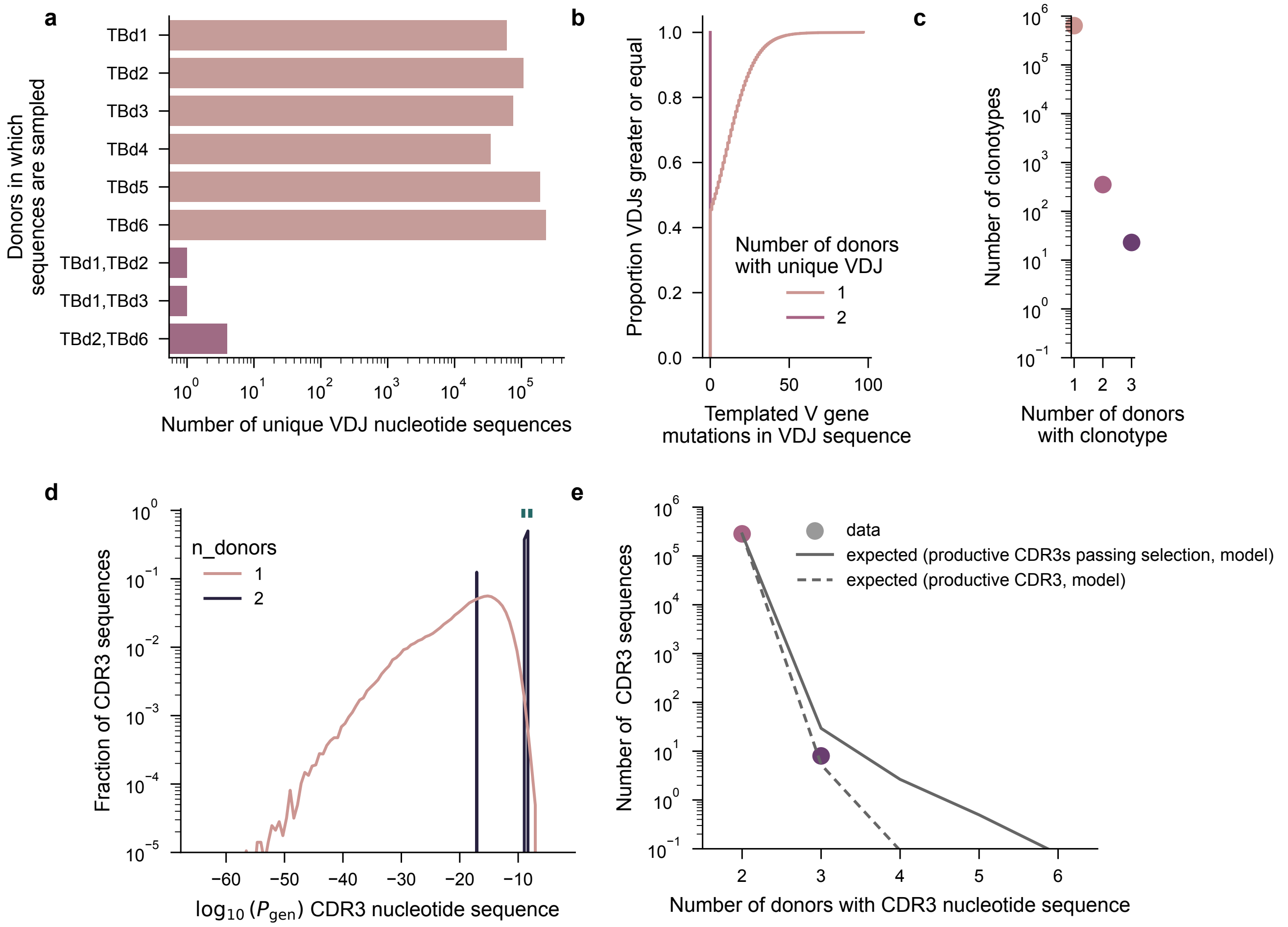

Fig. S9. Characteristics of VDJ sequences found in multiple donors. (a) Number of unique nucleotide VDJ sequences as a function of the donors they were found in. (b) Distribution of the number of hypermutations in the templated portion of the IGHV gene as a function of the number of donors a VDJ sequence was found in. (c) Number of shared "clonotypes" in our data, where a clonotype is taken to be a sequence with an identical germline V gene, J gene, and CDR3 amino acid sequence. (d) Recombination probabilities for the CDR3 nucleotide sequence as a function of the number of donors the CDR3 was found in. Green dashes denote the CDR3's associated with the VDJ sequences shared between multiple donors. (e) Number of shared CDR3s in our data compared to the number expected under given the overall distribution of recombination probabilities shown in (d). The two models are described in detail in **Section** D of the SI Appendix.

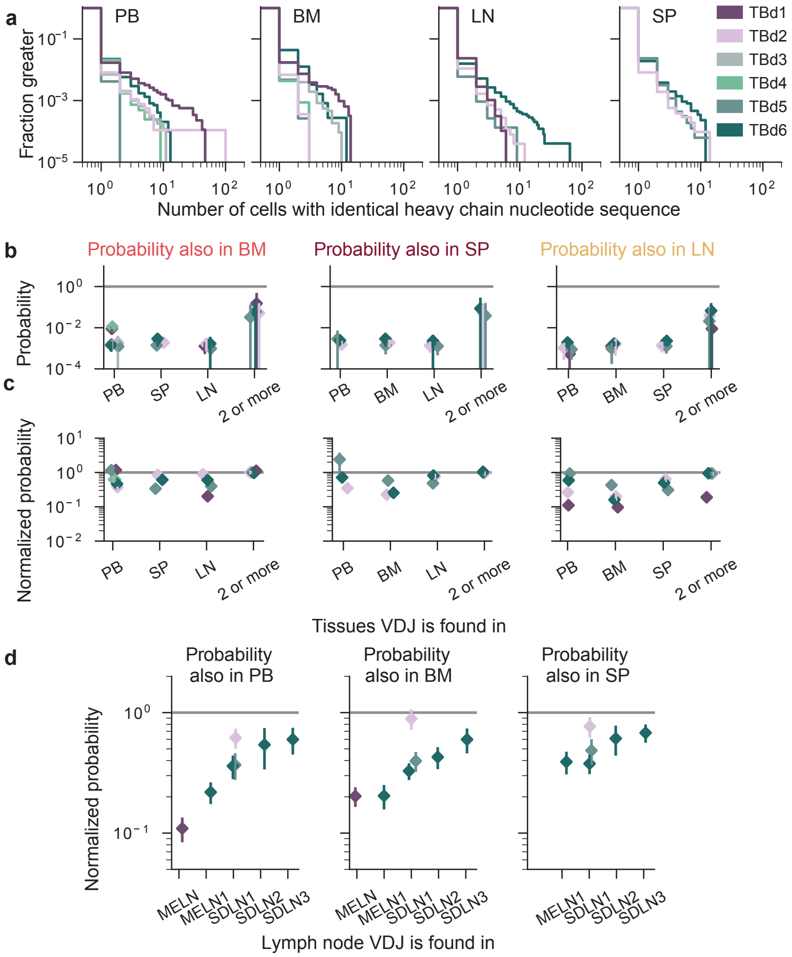

Fig. S10. Clonal expansion and hypermutation distributions across tissues and localization statistics of shared B cell clones. (a) To avoid conflation of VDJ dispersion in the ambient and true B cell expansion, the distributions are derived from droplets containing a single VDJ not found in ambient droplets and a high-quality B single B cell transcriptome. (b) Probability that a B cell clone is found in the bone marrow, spleen and lymph nodes, given its VDJ has been sampled in a different tissue, analogously to in **Fig. 6a** of the Main Text. Colors as in panel (a). (c) The normalized probability of appearance in the same tissues, analogously to **Fig. 6b** of the Main Text. (d) Normalized probability that a B cell clone is found in the peripheral blood, bone marrow, or spleen conditional on it having been sampled in a specific lymph node. MELN refers to the mixture of three different mesenteric lymph nodes from TBd1; MELN1 refers to the single mesenteric lymph node from TBd6, and SDLN1-3 refer to the three individual supradiaphragmatic lymph nodes from TBd6.

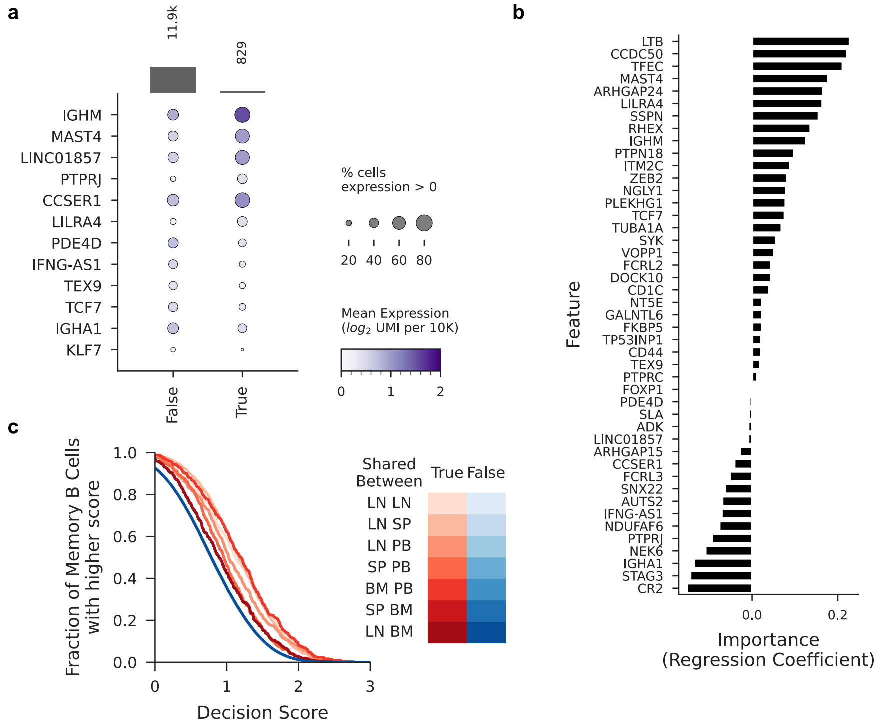

Fig. S11. Gene expression signatures of shared memory B cells. (a) Dotplot showing the gene expression differences conditional on whether a memory B cell is shared between Lymph Nodes and Spleen in TBd6 with bars showing total numbers of cells in each group (b) Feature importances for a logistic regression classifier constructed to predict whether or not a memory B cell is shared between Lymph Nodes and Spleen for all donors; features were selected from differential expression analysis on TBd6 shown in (a) (c) Distribution of decision scores of that same logistic regression for all memory B cells from all donors colored by whether or not a memory B cell is detected as shared between given tissue pair. Note that all distributions of memory B cells that are unshared (blue lines) overlap. For additional detail see **Section** E.3 of the SI Appendix.

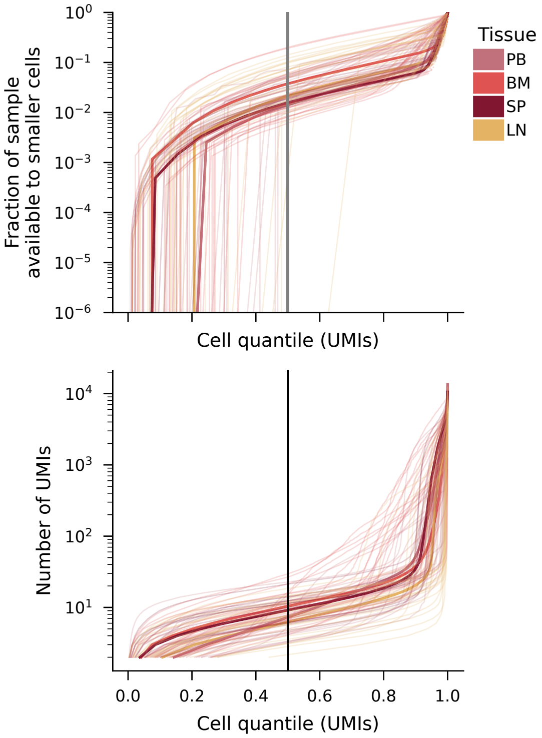

Fig. S12. Sample depletion by antibody-secreting cells. Number of UMIs (bottom) and cumulative fraction of sample available (top) as a function of cell IGH expression quantile. Thin lines correspond to individual libraries, thick lines represent the aggregate distributions for all samples of a certain tissue. Individual samples and aggregate distributions are colored by tissue of origin.

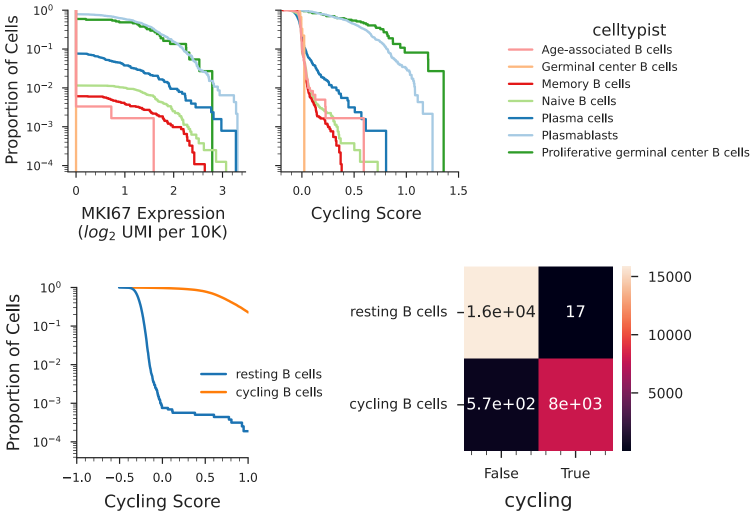

Fig. S13. Cell cycle annotation. (Top) Distributions of MKI67 expression (left) and Cell Cycling scores (right) colored by celltypist labels. (Bottom) Distributions of Cell Cycling Scores for *in vitro* cycling B cells and resting B cells for Ref. (7) (left) and confusion matrix showing the ability of cycling score to distinguish between cycling and non-cycling B cells from at a score cutoff of 0.01

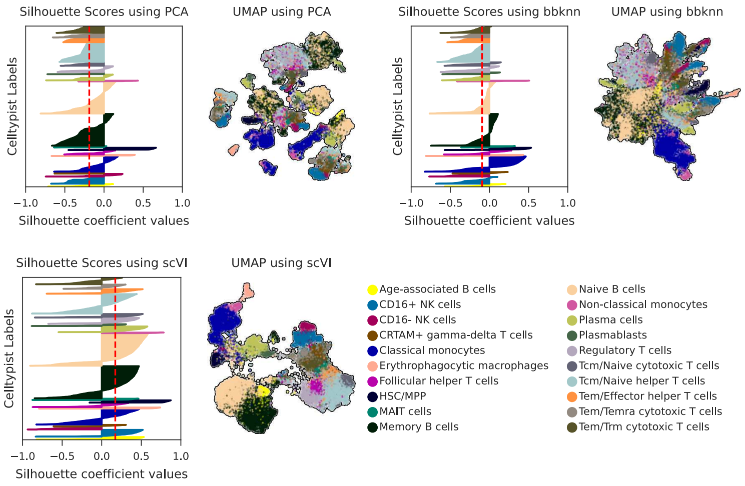

Fig. S14. Silhouette scores upon batch correction/integration. Silhouette scores and qualitative clustering performance on the TICA dataset plus ours improves when using scVI compared to PCA or BBKNN. Each pair of panels (top left, top right, and bottom shows the results of calculating the UMAP based on the nearest neighbors graphs from PCA, BBKNN, and scVI, respectively. The left panel shows distributions of silhouette scores for clusters in UMAP space, where clusters are cell types as defined by the celltypist prediction `Immune_All_low`. The right panels are the UMAPs colored by these cell type labels showing qualitative improvement in clustering accuracy across different samples. Dataset was downsampled and re-balanced to 500 cells per batch key and cell types with less than 250 representatives were removed for visualization purposes

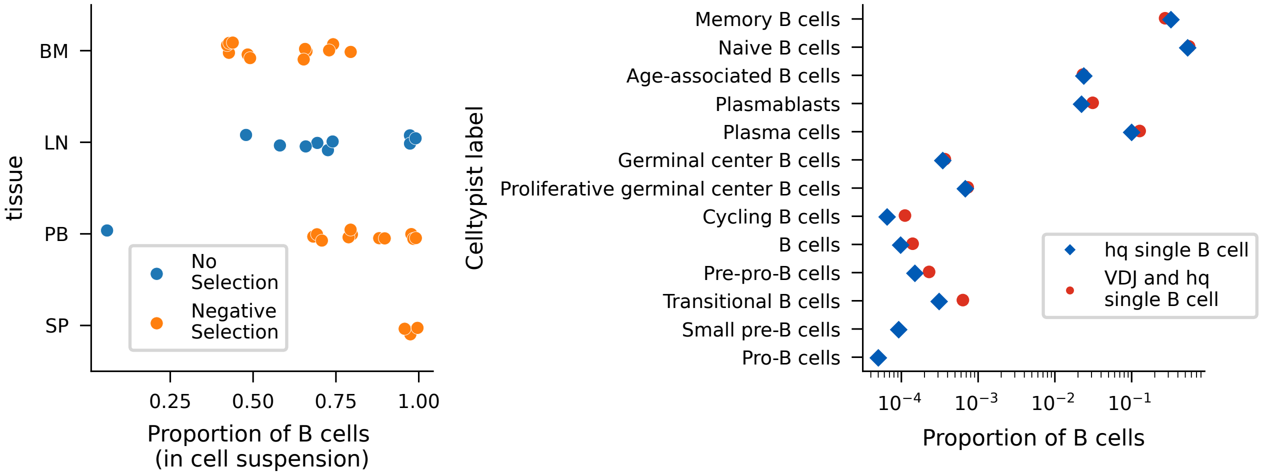

Fig. S15. Biases in relative abundances of B cell types during sampling. (Left) Fraction of all cells that are B cells in each cell suspension. Samples are colored by whether or not a B cell purification was performed. (Right) Relative abundances of B cell subsets when either conditioned on having an associated VDJ or not. Cell type labels are from the celltypist Immune_All_Low model.

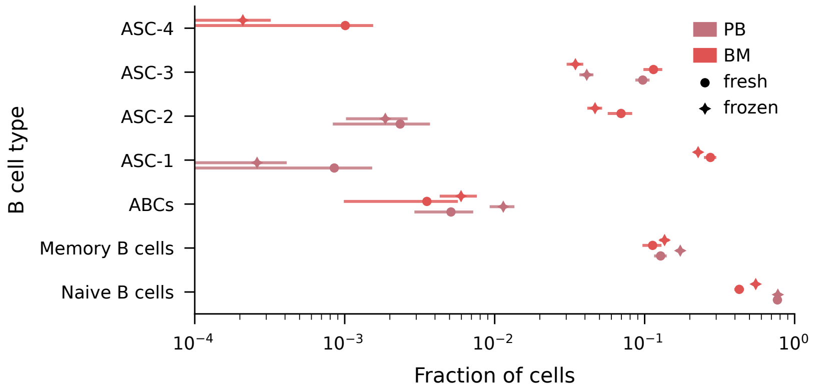

Fig. S16. Biases in cell subsets due to the freeze-thaw procedure Proportion of high-quality singlet non-ambient B cells in each B cell subset of the peripheral blood and bone marrow of TBd3. Error bars denote 95% confidence intervals obtained under the assumption of binomial sampling.

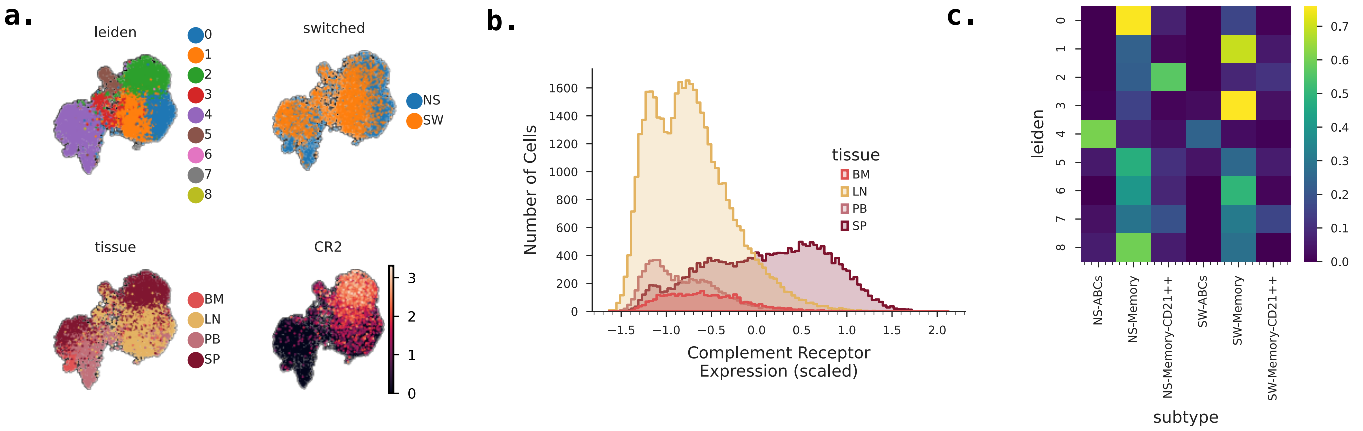

Fig. S17. Memory B cell phenotypes. (a) UMAPs showing categorical axes of variation in memory B cell subsets (b) Distributions of complement receptor expression derived scores for memory B cells colored by tissue (c) Confusion matrix showing the agreement between leiden-based data-dependent clustering and heuristic clustering

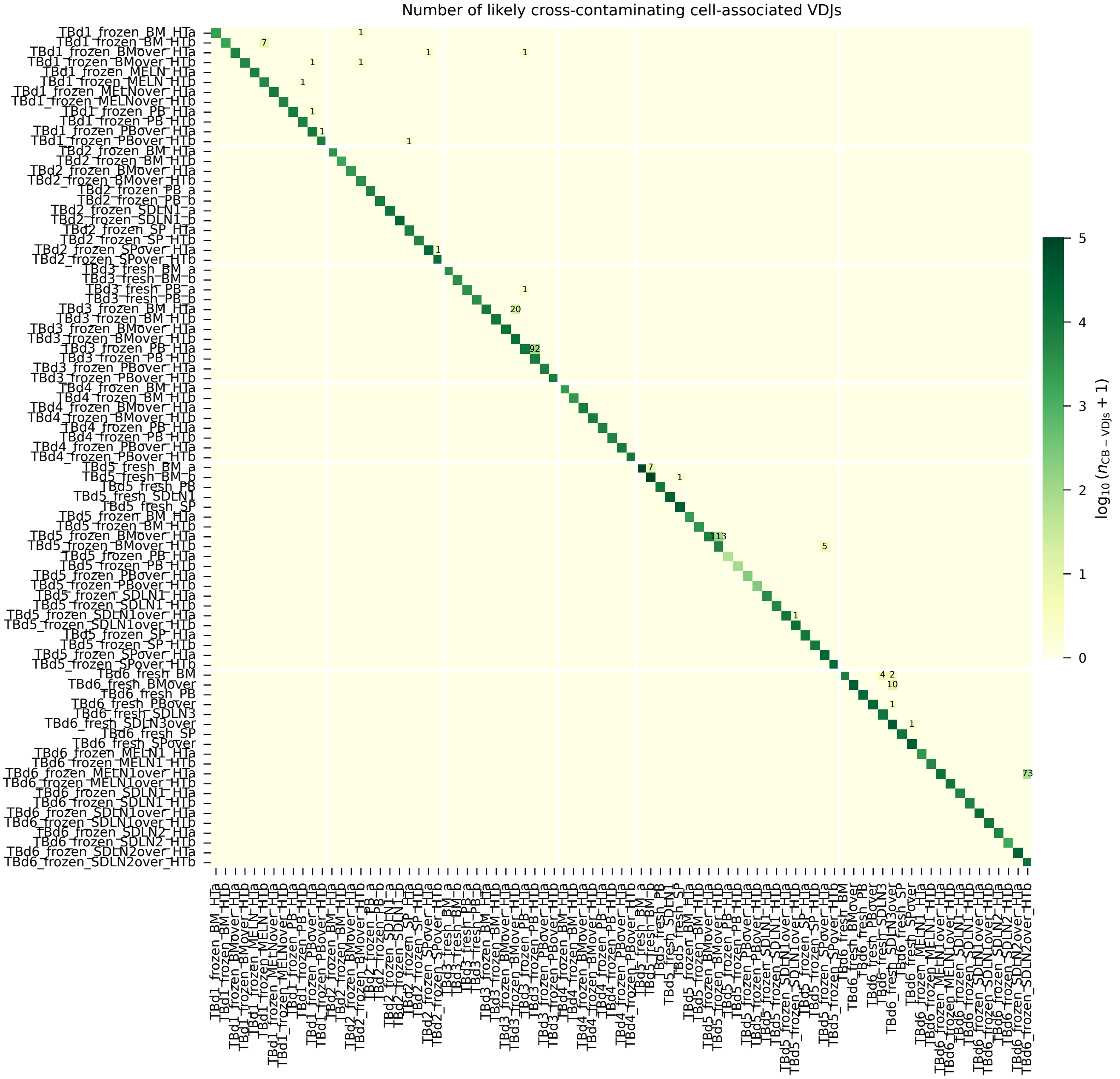

Fig. S18. Distribution of putative cross-contaminating heavy chains. Heatmap is colored by the logarithm of the total number of likely cross-contaminating cell barcode-VDJs for each pair of samples, and by the logarithm of the total number of private cell barcode-VDJs on the diagonal. The off-diagonal elements are annotated with the number of putative cross-contaminating VDJs.

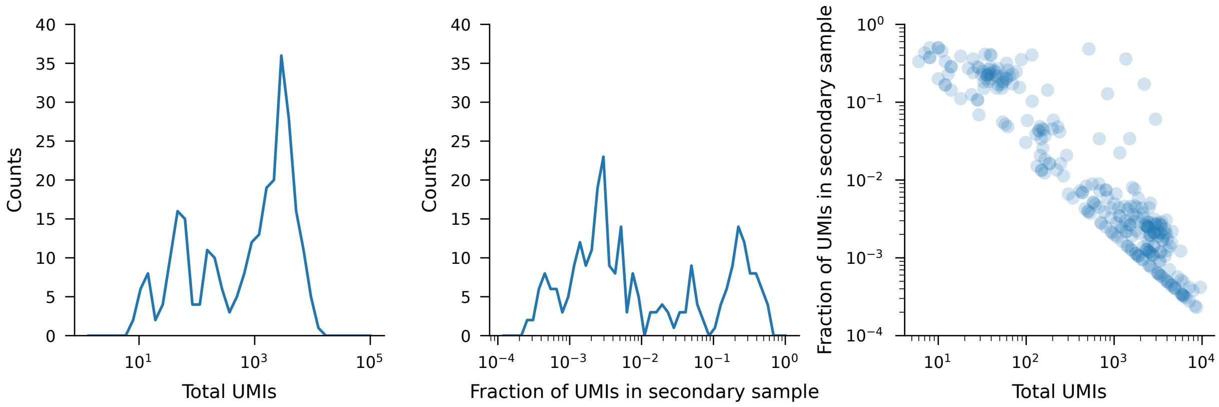

Fig. S19. Distribution of putative cross-contaminating heavy chains among the pair of samples between which they are shared. (Left) Distribution of the total number of UMIs associated with the shared cell barcode-VDJ across the pair of samples. (Center) Distribution of the fraction of UMIs found in the sample with the smaller number of UMIs belonging to the cell barcode-VDJ pair. (Right) Scatterplot showing the two quantities for each shared cell barcode-VDJ event.

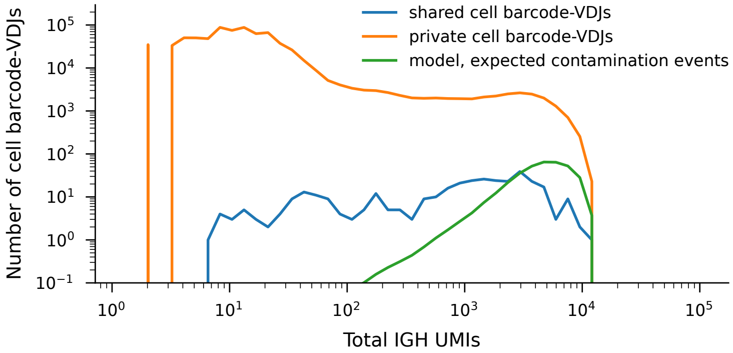

Fig. S20. Heavy chain UMI abundance among shared cell barcode-VDJs and private cell barcode-VDJs among samples involved in any shared cell barcode-VDJ events.

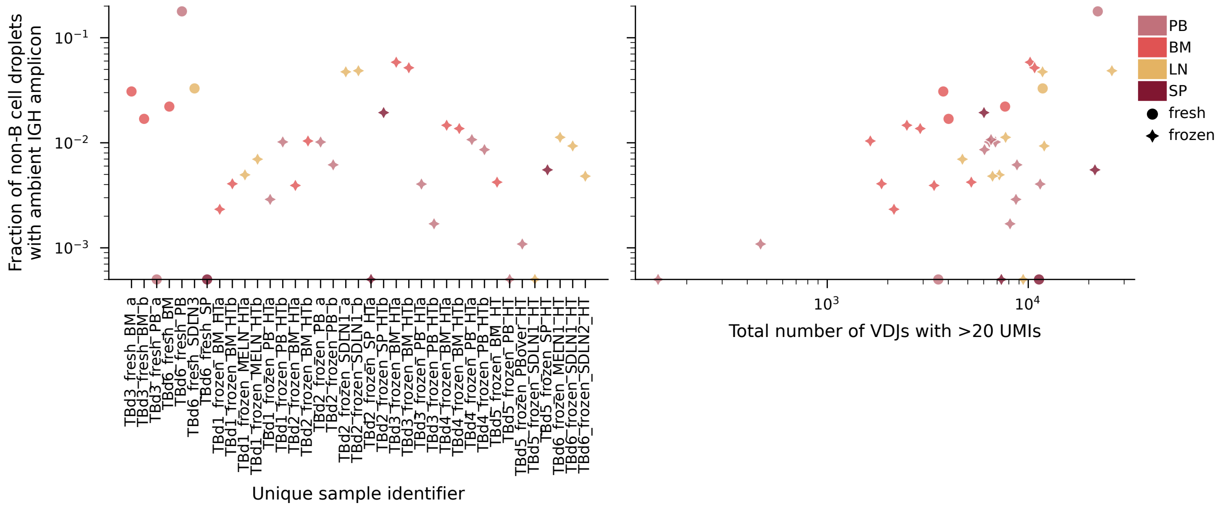

Fig. S21. Abundance of ambient VDJs. Samples are represented by a symbol corresponding to whether they were prepared fresh or first frozen and stored in liquid nitrogen, and colored by their tissue of origin. Points on the x-axis represent samples in which no VDJ transcripts were found in droplets containing high quality B cells.

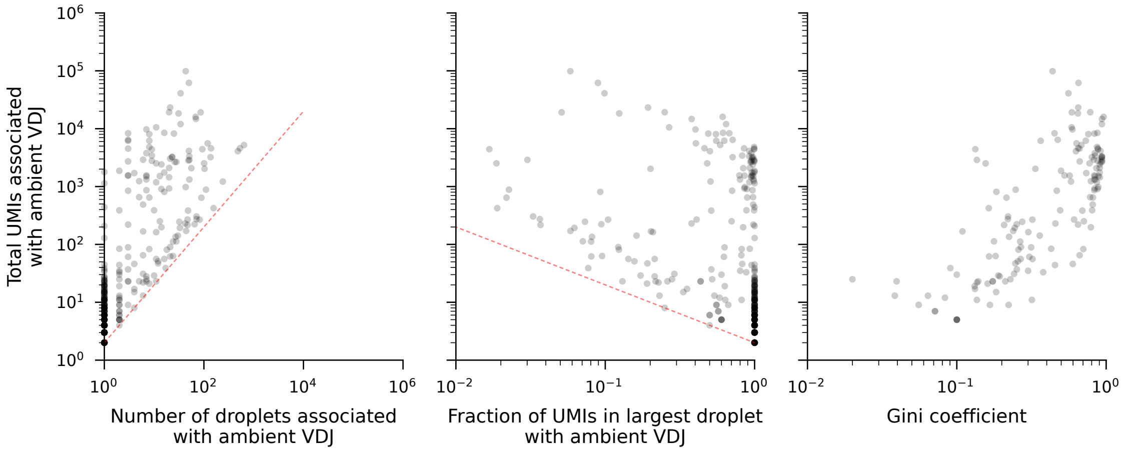

Fig. S22. Distribution of ambient VDJs among droplets (Left) Number of droplets and total UMIs associated with ambient VDJ. Each point represents a unique VDJ sequence associated with at least one droplet containing a high quality single non-B cell transcriptome. Red line denotes expectation under well mixed model: given the microscopic sizes of each droplet ($\sim 100\mathrm{pL}$) and their enormous number in the overall volume ($\sim 100\mu\mathrm{L}$), the expected number of UMIs per droplet ranges from $\sim 10^{-6}$ to $\sim 10^{-2}$. Thus, under the well-mixed model, the probability of detecting more than the filter threshold 2 UMIs is microscopically small for the vast majority of VDJs. (Center) Fraction of UMIs in largest droplet with ambient VDJ. Dashed red line once again denotes filter threshold, expected under well-mixed model. (Right) Gini coefficient for ambient VDJs detected in more than one droplet.

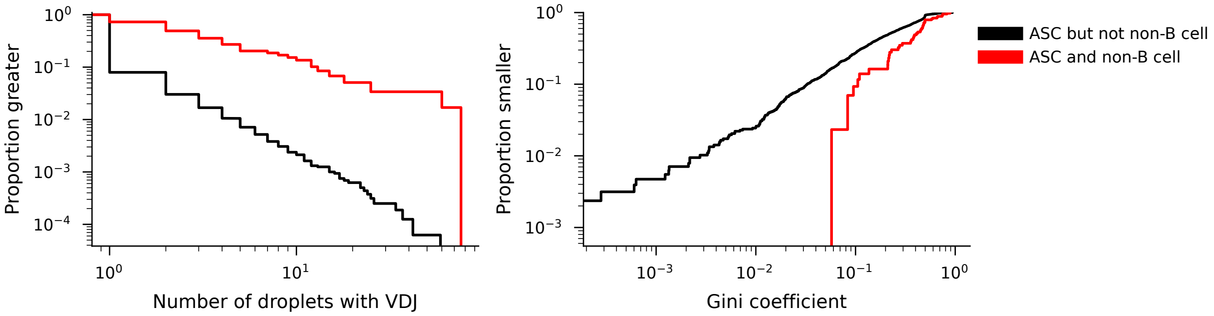

Fig. S23. Distribution of ASC-associated VDJs among droplets (Left) Distribution of the number of high-quality B cell droplets containing an ASC-associated VDJ for ASC-associated VDJs that either were or were not also associated with a non-B cell. (Right) Gini coefficient for ambient VDJs detected in more than one droplet.

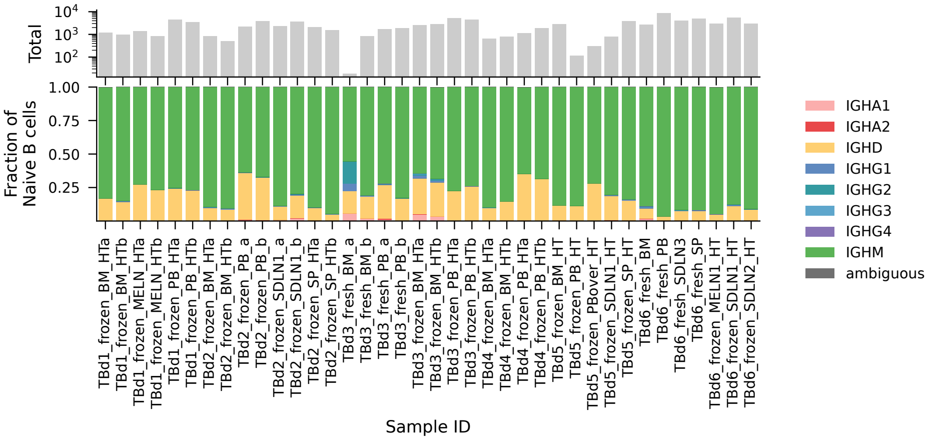

Fig. S24. Presence of rare class-switched ambient contaminants within droplets with Naive B cell gene expression. (Top) Total number of single high quality B cell droplets with a VDJ multiplicity of 1 and a Naive GEX profile. (Bottom) The distribution non-ambiguous IGHC gene assignments among VDJs associated with the same cells. Each bar corresponds to a unique GEX sample.

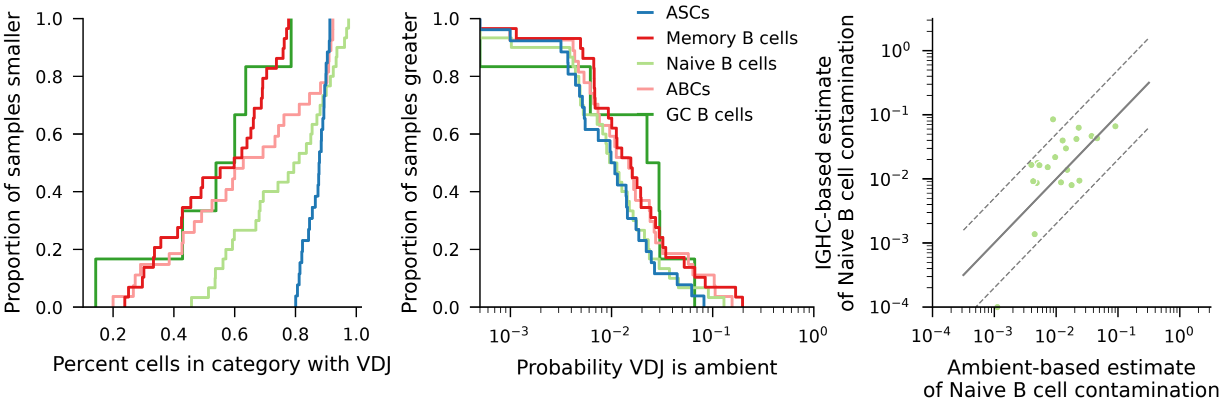

Fig. S25. Misidentification of ambient VDJs with GEX signatures (Left) Distribution of rates at which a GEX profile has any identified VDJ transcripts in the dataset for each B cell subtype. (Center) The distribution of probabilities that the identified VDJ is ambient in origin for each B cell subtype. (Right) Concordance between Naive B cell droplet contamination rates from the ambient estimated based on the rates of VDJs are found associated with non-B cells (x-axis), or are class-switched (y-axis, points along the axes correspond to zeros). Each point corresponds to a unique GEX sample. The full grey line represents perfect agreement between the two estimates, and the region between the dashed grey lines corresponds to the two estimates being within a factor of 5 of each other.

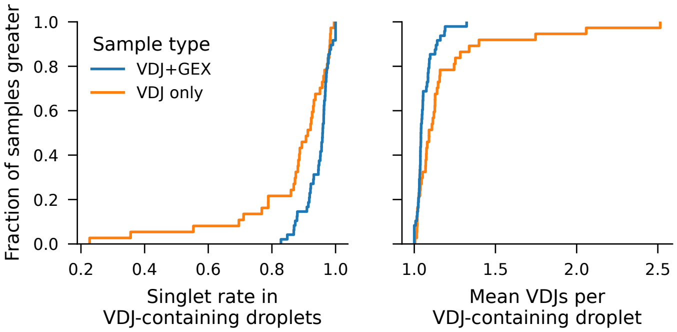

Fig. S26. Rates of VDJ multiplicity in droplets (Left) Distribution of the fraction of VDJ-containing droplets that contain a single unique heavy chain cell call ("VDJ"). Samples are stratified by whether or not they were loaded at 20 000 cells per lane ("VDJ + GEX"), or 80 000 cells per lane ("VDJ only"). (Right) Average number of VDJ sequences in VDJ containing droplet.

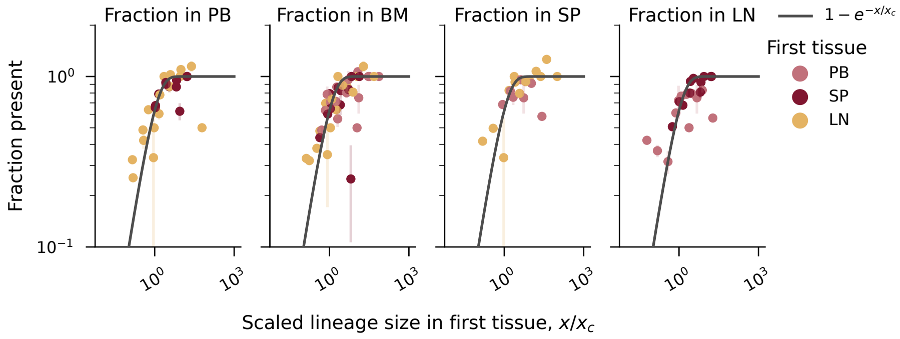

Fig. S27. Sharing of lineages between pairs of tissues. Fraction of lineages found at given frequency in the peripheral blood, lymph node, or spleen (different colors) that are present in a second tissue. Each panel corresponds to a different second tissue. Data points were binned by frequency in the first tissue, and scaled by x_c, as described above. The fraction present has been calculated as described in Eq. 9. Grey line represents theoretical expectation under a model of independent exit of cells from the first tissue, see Eq. 12.

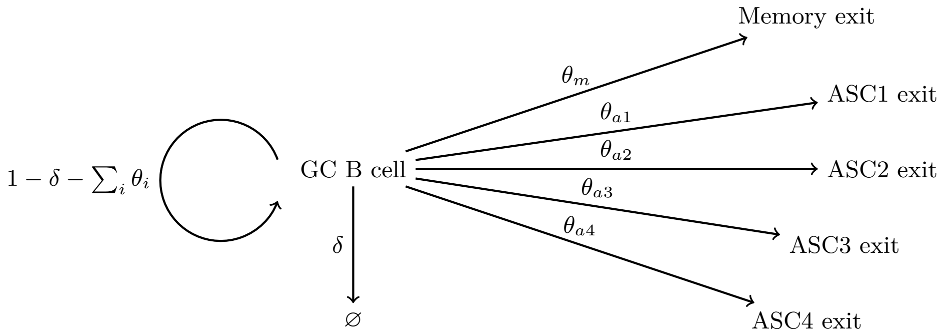

Fig. S28. Schematic illustrating a model of a memoryless differentiation process within an active germinal center. The per-LZ/DZ cycle probabilities of each of the events are denoted by the symbols accompanying each arrow.

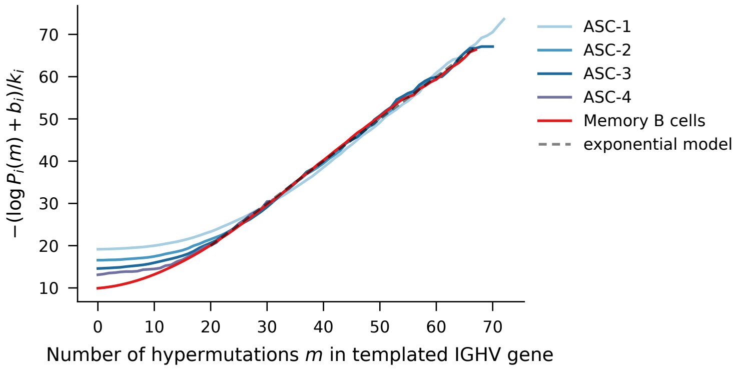

Fig. S29. Scaled logarithm of the complementary CDF of the hypermutation number distribution. Eq. 24 is indicated by the dashed grey line. Parameters k_i and C_i for each cell type were inferred inferred by least-squares linear regression of the logarithm of the complementary CDF for $m \geq 20$. Portions of the CDF supported by fewer than 20 cells have been truncated.

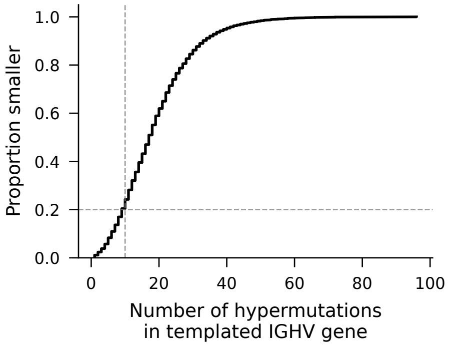

Fig. S30. Distribution of hypermutation numbers in all cells found in lineages with at least 3 sampled unique VDJs Dotted lines indicate point at which the ratio of Memory B cell GC exit rates and ASC GC exit rates appears to stabilize.

Donor ID	Tissue	Anatomical Location
TBd1	peripheral blood	-
	bone marrow	vertebral body
	lymph node	mesenteric
TBd2	peripheral blood	-
	bone marrow	vertebral body
	lymph node	supradiaphragmatic
	spleen	-
TBd3	peripheral blood	-
	bone marrow	vertebral body
TBd4	peripheral blood	-
	bone marrow	vertebral body
TBd5	peripheral blood	-
	bone marrow	vertebral body
	lymph node	supradiaphragmatic
	spleen	-
TBd6	peripheral blood	-
	bone marrow	vertebral body
	lymph node	supradiaphragmatic, mesenteric
	spleen	-

Table S1. List of all tissues used in this study and their anatomical locations.

Cell Suspension	Unique Sample ID	Technical Sibling	GEX	B cell enriched
TBd1_frozen_BM	TBd1_frozen_BM_HTa	TBd1_frozen_BM_HTb	✓	✓
TBd1_frozen_BM	TBd1_frozen_BM_HTb	TBd1_frozen_BM_HTa	✓	✓
TBd1_frozen_BM	TBd1_frozen_BMover_HTa	TBd1_frozen_BMover_HTb		✓
TBd1_frozen_BM	TBd1_frozen_BMover_HTb	TBd1_frozen_BMover_HTa		✓
TBd1_frozen_MELN	TBd1_frozen_MELN_HTa	TBd1_frozen_MELN_HTb	✓	
TBd1_frozen_MELN	TBd1_frozen_MELN_HTb	TBd1_frozen_MELN_HTa	✓	
TBd1_frozen_MELN	TBd1_frozen_MELNover_HTa	TBd1_frozen_MELNover_HTb		
TBd1_frozen_MELN	TBd1_frozen_MELNover_HTb	TBd1_frozen_MELNover_HTa		
TBd1_frozen_PB	TBd1_frozen_PB_HTa	TBd1_frozen_PB_HTb	✓	✓
TBd1_frozen_PB	TBd1_frozen_PB_HTb	TBd1_frozen_PB_HTa	✓	✓
TBd1_frozen_PB	TBd1_frozen_PBover_HTa	TBd1_frozen_PBover_HTb		✓
TBd1_frozen_PB	TBd1_frozen_PBover_HTb	TBd1_frozen_PBover_HTa		✓
TBd2_frozen_BM	TBd2_frozen_BM_HTa	TBd2_frozen_BM_HTb	✓	✓
TBd2_frozen_BM	TBd2_frozen_BM_HTb	TBd2_frozen_BM_HTa	✓	✓
TBd2_frozen_BM	TBd2_frozen_BMover_HTa	TBd2_frozen_BMover_HTb		✓
TBd2_frozen_BM	TBd2_frozen_BMover_HTb	TBd2_frozen_BMover_HTa		✓
TBd2_frozen_SDLN1	TBd2_frozen_SDLN1_a		✓	
TBd2_frozen_SDLN1	TBd2_frozen_SDLN1_b		✓	
TBd2_frozen_PB	TBd2_frozen_PB_a		✓	✓
TBd2_frozen_PB	TBd2_frozen_PB_b		✓	✓
TBd2_frozen_SP	TBd2_frozen_SP_HTa	TBd2_frozen_SP_HTb	✓	✓
TBd2_frozen_SP	TBd2_frozen_SP_HTb	TBd2_frozen_SP_HTa	✓	✓
TBd2_frozen_SP	TBd2_frozen_SPover_HTa	TBd2_frozen_SPover_HTb		✓
TBd2_frozen_SP	TBd2_frozen_SPover_HTb	TBd2_frozen_SPover_HTa		✓
TBd3_fresh_BM	TBd3_fresh_BM_a		✓	✓
TBd3_fresh_BM	TBd3_fresh_BM_b		✓	✓
TBd3_frozen_BM	TBd3_frozen_BM_HTa	TBd3_frozen_BM_HTb	✓	✓
TBd3_frozen_BM	TBd3_frozen_BM_HTb	TBd3_frozen_BM_HTa	✓	✓
TBd3_frozen_BM	TBd3_frozen_BMover_HTa	TBd3_frozen_BMover_HTb		✓
TBd3_frozen_BM	TBd3_frozen_BMover_HTb	TBd3_frozen_BMover_HTa		✓
TBd3_fresh_PB	TBd3_fresh_PB_a		✓	✓
TBd3_fresh_PB	TBd3_fresh_PB_b		✓	✓
TBd3_frozen_PB	TBd3_frozen_PB_HTa	TBd3_frozen_PB_HTb	✓	✓
TBd3_frozen_PB	TBd3_frozen_PB_HTb	TBd3_frozen_PB_HTa	✓	✓
TBd3_frozen_PB	TBd3_frozen_PBover_HTa	TBd3_frozen_PBover_HTb		✓
TBd3_frozen_PB	TBd3_frozen_PBover_HTb	TBd3_frozen_PBover_HTa		✓
TBd4_frozen_BM	TBd4_frozen_BM_HTa	TBd4_frozen_BM_HTb	✓	✓
TBd4_frozen_BM	TBd4_frozen_BM_HTb	TBd4_frozen_BM_HTa	✓	✓
TBd4_frozen_BM	TBd4_frozen_BMover_HTa	TBd4_frozen_BMover_HTb		✓
TBd4_frozen_BM	TBd4_frozen_BMover_HTb	TBd4_frozen_BMover_HTa		✓
TBd4_frozen_PB	TBd4_frozen_PB_HTa	TBd4_frozen_PB_HTb	✓	✓
TBd4_frozen_PB	TBd4_frozen_PB_HTb	TBd4_frozen_PB_HTa	✓	✓
TBd4_frozen_PB	TBd4_frozen_PBover_HTa	TBd4_frozen_PBover_HTb		✓
TBd4_frozen_PB	TBd4_frozen_PBover_HTb	TBd4_frozen_PBover_HTa		✓
TBd5_fresh_BM	TBd5_fresh_BM_a			✓
TBd5_fresh_BM	TBd5_fresh_BM_b			✓
TBd5_frozen_BM	TBd5_frozen_BM_HTa	TBd5_frozen_BM_HTb	✓	✓
TBd5_frozen_BM	TBd5_frozen_BM_HTb	TBd5_frozen_BM_HTa	✓	✓
TBd5_frozen_BM	TBd5_frozen_BMover_HTa	TBd5_frozen_BMover_HTb		✓
TBd5_frozen_BM	TBd5_frozen_BMover_HTb	TBd5_frozen_BMover_HTa		✓
TBd5_fresh_SDLN1	TBd5_fresh_SDLN1			
TBd5_frozen_SDLN1	TBd5_frozen_SDLN1_HTa	TBd5_frozen_SDLN1_HTb	✓	
TBd5_frozen_SDLN1	TBd5_frozen_SDLN1_HTb	TBd5_frozen_SDLN1_HTa	✓	
TBd5_frozen_SDLN1	TBd5_frozen_SDLN1over_HTa	TBd5_frozen_SDLN1over_HTb		
TBd5_frozen_SDLN1	TBd5_frozen_SDLN1over_HTb	TBd5_frozen_SDLN1over_HTa		
TBd5_fresh_PB	TBd5_fresh_PB			✓
TBd5_fresh_SP	TBd5_fresh_SP			✓
TBd5_frozen_PB	TBd5_frozen_PB_HTa	TBd5_frozen_PB_HTb	✓	

Cell Suspension	Unique Sample ID	Technical Sibling	GEX	B cell enriched
TBd5_frozen_PB	TBd5_frozen_PB_HTb	TBd5_frozen_PB_HTa	✓	
TBd5_frozen_PB	TBd5_frozen_PBover_HTa	TBd5_frozen_PBover_HTb	✓	
TBd5_frozen_PB	TBd5_frozen_PBover_HTb	TBd5_frozen_PBover_HTa	✓	
TBd5_frozen_SP	TBd5_frozen_SP_HTa	TBd5_frozen_SP_HTb	✓	✓
TBd5_frozen_SP	TBd5_frozen_SP_HTb	TBd5_frozen_SP_HTa	✓	✓
TBd5_frozen_SP	TBd5_frozen_SPover_HTa	TBd5_frozen_SPover_HTb		✓
TBd5_frozen_SP	TBd5_frozen_SPover_HTb	TBd5_frozen_SPover_HTa		✓
TBd6_fresh_BM	TBd6_fresh_BM		✓	✓
TBd6_fresh_BM	TBd6_fresh_BMover			✓
TBd6_fresh_SDLN3	TBd6_fresh_SDLN3		✓	
TBd6_fresh_SDLN3	TBd6_fresh_SDLN3over			
TBd6_frozen_MELN1	TBd6_frozen_MELN1_HTa	TBd6_frozen_MELN1_HTb	✓	
TBd6_frozen_MELN1	TBd6_frozen_MELN1_HTb	TBd6_frozen_MELN1_HTa	✓	
TBd6_frozen_MELN1	TBd6_frozen_MELN1over_HTa	TBd6_frozen_MELN1over_HTb		
TBd6_frozen_MELN1	TBd6_frozen_MELN1over_HTb	TBd6_frozen_MELN1over_HTa		
TBd6_frozen_SDLN1	TBd6_frozen_SDLN1_HTa	TBd6_frozen_SDLN1_HTb	✓	
TBd6_frozen_SDLN1	TBd6_frozen_SDLN1_HTb	TBd6_frozen_SDLN1_HTa	✓	
TBd6_frozen_SDLN1	TBd6_frozen_SDLN1over_HTa	TBd6_frozen_SDLN1over_HTb		
TBd6_frozen_SDLN1	TBd6_frozen_SDLN1over_HTb	TBd6_frozen_SDLN1over_HTa		
TBd6_frozen_SDLN2	TBd6_frozen_SDLN2_HTa	TBd6_frozen_SDLN2_HTb	✓	
TBd6_frozen_SDLN2	TBd6_frozen_SDLN2_HTb	TBd6_frozen_SDLN2_HTa	✓	
TBd6_frozen_SDLN2	TBd6_frozen_SDLN2over_HTa	TBd6_frozen_SDLN2over_HTb		
TBd6_frozen_SDLN2	TBd6_frozen_SDLN2over_HTb	TBd6_frozen_SDLN2over_HTa		
TBd6_fresh_PB	TBd6_fresh_PB		✓	✓
TBd6_fresh_PB	TBd6_fresh_PBover			✓
TBd6_fresh_SP	TBd6_fresh_SP		✓	✓
TBd6_fresh_SP	TBd6_fresh_SPover			✓

Table S2. Enumeration of all sequencing libraries analyzed in this study. VDJ libraries were prepared and analyzed for all samples listed. Samples have HT in their name if they were generated on the 10X Chromium X Controller; all other samples were generated on the 10X Chromium Controller. Unless designated as technical siblings, samples of the same tissue represent separate samples of the live cell suspensions noted in the column. Technical siblings represent the two equal volume parts of the same emulsion, generated on the 10X Chromium X Controller using the 5′ high throughput kit. These were typically processed, indexed, and sequenced separately. We did not split the emulsion in samples generated on the standard throughput kit on the Chromium Controller. "B cell enriched" denotes that negative selection for B cells was performed on the sample.

Exit state	Inferred slope, k_i	Per-cycle exit rate ε_i
ASC-1	0.10	0.030
ASC-2	0.10	0.029
ASC-3	0.11	0.033
ASC-4	0.10	0.029
Memory B cells	0.14	0.041

Table S3. Inferred per-cycle exit rates for the model implied in Fig. S28.

References

1. TS Consortium*, et al., The tabula sapiens: A multiple-organ, single-cell transcriptomic atlas of humans. *Science* **376**, eabl4896 (2022).
2. W Shi, et al., Transcriptional profiling of mouse B cell terminal differentiation defines a signature for antibody-secreting plasma cells. *Nat. Immunol.* **16**, 663–673 (2015).
3. SJ Fleming, JC Marioni, M Babadi, Cellbender remove-background: a deep generative model for unsupervised removal of background noise from scrna-seq datasets. *BioRxiv* **791699** (2019).
4. C Domínguez Conde, et al., Cross-tissue immune cell analysis reveals tissue-specific features in humans. *Science* **376**, eabl5197 (2022).
5. Pan-immune-dataset (https://www.tissueimmunecellatlas.org/#datasets) (2022) Accessed: 2023-07-17.
6. SL Wolock, R Lopez, AM Klein, Scrublet: computational identification of cell doublets in single-cell transcriptomic data. *Cell systems* **8**, 281–291 (2019).
7. M Swift, F Horns, SR Quake, Lineage tracing reveals fate bias and transcriptional memory in human B cells. *Life Sci. Alliance* **6** (2023).
8. R Lopez, J Regier, MB Cole, MI Jordan, N Yosef, Deep generative modeling for single-cell transcriptomics. *Nat. methods* **15**, 1053–1058 (2018).
9. K Polański, et al., BBKNN: fast batch alignment of single cell transcriptomes. *Bioinformatics* **36**, 964–965 (2020).
10. MD Luecken, et al., Benchmarking atlas-level data integration in single-cell genomics. *Nat. methods* **19**, 41–50 (2022).
11. I Cvijovic, ER Jerison, SR Quake, Reference-free germline immunoglobulin allele discovery from B cell receptor sequencing data. *bioRxiv* **568681** (2023).
12. JQ Zhou, SH Kleinstein, Cutting edge: Ig H chains are sufficient to determine most B cell clonal relationships. *The J. Immunol.* **203**, 1687–1692 (2019).
13. C Soto, et al., High frequency of shared clonotypes in human B cell receptor repertoires. *Nature* **566**, 398–402 (2019).
14. Z Sethna, Y Elhanati, CG Callan, AM Walczak, T Mora, OLGA: fast computation of generation probabilities of B- and T-cell receptor amino acid sequences and motifs. *Bioinformatics* **35**, 2974–2981 (2019).
15. Y Elhanati, Z Sethna, CG Callan Jr, T Mora, AM Walczak, Predicting the spectrum of TCR repertoire sharing with a data-driven model of recombination. *Immunol. Rev.* **284**, 167–179 (2018).
16. R Pavri, MC Nussenzweig, Aid targeting in antibody diversity. *Adv. immunology* **110**, 1–26 (2011).
17. JM Di Noia, MS Neuberger, Molecular mechanisms of antibody somatic hypermutation. *Annu. Rev. Biochem.* **76**, 1–22 (2007).
18. GD Victora, MC Nussenzweig, Germinal centers. *Annu. review immunology* **40**, 413–442 (2022).
